# Netrins and Wnts Function Redundantly to Regulate Antero-Posterior and Dorso-Ventral Guidance in *C. elegans*

**DOI:** 10.1371/journal.pgen.1004381

**Published:** 2014-06-05

**Authors:** Naomi Levy-Strumpf, Joseph G. Culotti

**Affiliations:** 1 Lunenfeld-Tanenbaum Research Institute of Mount Sinai Hospital, Toronto, Ontario, Canada; 2 Department of Molecular Genetics, University of Toronto, Toronto, Ontario, Canada; Harvard University, United States of America

## Abstract

Guided migrations of cells and developing axons along the dorso-ventral (D/V) and antero-posterior (A/P) body axes govern tissue patterning and neuronal connections. In *C. elegans*, as in vertebrates, D/V and A/P graded distributions of UNC-6/Netrin and Wnts, respectively, provide instructive polarity information to guide cells and axons migrating along these axes. By means of a comprehensive genetic analysis, we found that simultaneous loss of Wnt and Netrin signaling components reveals previously unknown and unexpected redundant roles for Wnt and Netrin signaling pathways in both D/V and A/P guidance of migrating cells and axons in *C. elegans*, as well as in processes essential for organ function and viability. Thus, in addition to providing polarity information for migration along the axis of their gradation, Wnts and Netrin are each able to guide migrations orthogonal to the axis of their gradation. Netrin signaling not only functions redundantly with some Wnts, but also counterbalances the effects of others to guide A/P migrations, while the involvement of Wnt signaling in D/V guidance identifies Wnt signaling as one of the long sought mechanisms that functions in parallel to Netrin signaling to promote D/V guidance of cells and axons. These findings provide new avenues for deciphering how A/P and D/V guidance signals are integrated within the cell to establish polarity in multiple biological processes, and implicate broader roles for Netrin and Wnt signaling - roles that are currently masked due to prevalent redundancy.

## Author Summary

While ample information was gathered in past decades on identifying guidance cues and their downstream mediators, very little is known about how the information from multiple extracellular cues is integrated within the cell to generate normal patterning. Netrin and Wnt signaling pathways are both critical to multiple developmental processes and play key roles in normal development as well as in malignancies. The UNC-6/Netrin guidance cue has a conserved role in guiding cell and growth cone migrations along the dorso-ventral axis, whereas Wnts are critical for determining polarity and guidance along the antero-posterior axis. In this study we show that these two signaling pathways function redundantly in both antero-posterior and dorso-ventral guidance as well as in processes essential for viability. Furthermore, we demonstrate that a fine balance between Wnt and Netrin signaling pathways is critical for proper polarity establishment and identify Wnt signaling as one of the long sought mechanisms that signal in parallel to Netrin to promote dorso-ventral guidance of cells and axons in *Caenorhabditis elegans*. These findings pave the way to unraveling the broader roles of Wnt and Netrin signaling pathways and provide a conceptually novel view of how antero-posterior and dorso-ventral guidance mechanisms are orchestrated.

## Introduction

Migrating cells and axons respond to a multitude of extracellular cues encountered along their migratory paths. These include secreted cues such as Netrins, which are known to guide migrating cells and axons along the D/V axis of invertebrates and the vertebrate spinal cord [1]–[3], and Wnts, which mediate guidance along the A/P axis [4]. While considerable advances have been made in identifying guidance cues and their downstream mediators, how information from multiple cues is integrated within the cell to enact normal migration patterns has yet to be fully elucidated.

To illuminate how a cell calculates the net response to multiple, sometimes additive, overlapping, or opposing inputs we decided to examine genetic interactions between UNC-6/Netrin and Wnt signaling components in the migration of cells and axons that navigate along the D/V or A/P axes of the body wall. In *C. elegans* a polarity-determining gradient of UNC-6/Netrin secreted by ventral sources of this guidance cue mediates apparent attraction of some migrating cells and growth cones toward the ventral side by signaling through the transmembrane receptor UNC-40/DCC, and also mediates apparent repulsion of other cells and growth cones away from the ventral side by signaling through the transmembrane receptor UNC-5 alone or together with UNC-40/DCC [1], [5]–[7]. This highly conserved instructive guidance system is critical for nervous system patterning in both vertebrates and invertebrates [2], [3], [5].

Wnts also play key roles in cell migration and axon guidance [4], [8], [9]. The *C. elegans* genome encodes five Wnt ligands (EGL-20, LIN-44, MOM-2, CWN-1, CWN-2), four frizzled receptors (LIN-17/Frizzled, MOM-5, MIG-1/Frizzled, CFZ-2) and a single RYK/Derailed receptor tyrosine kinase (LIN-18) [10]. Wnts, like UNC-6/Netrin, act as both short-range and long-range repellents or attractants, and can function instructively (i.e., their graded distribution determines polarity) as well as permissively (i.e., do not instruct, but are necessary for polarity) [8], [9], [11]–[16]. The Wnt binding protein MIG-14/Wntless is required in Wnt producing cells to facilitate Wnt secretion [17]. Wnt activity is further modulated by a number of inhibitors and activators [18]. One family of inhibitors is the Secreted Frizzled Related Proteins (SFRPs), which are soluble glycoproteins widely involved in embryonic development and homeostasis. SFRPs contain two functional domains: the cysteine rich domain (CRD) related to the extracellular portion of Frizzled Wnt receptors, and the Netrin related motif (NTR) defined by homology with Netrin-1. SFRPs can sequester Wnts thereby preventing Wnt ligand-receptor interactions [18].

Netrins and Wnts in *C. elegans* are well known for having a graded distribution along the D/V and A/P axes, respectively, and can provide polarity information for guiding migration up or down their respective gradients. Accordingly, *unc-6/netrin* mutants were originally found to affect D/V but not A/P migrations, whereas *wnt* mutants were originally found to affect A/P but not D/V migrations [1], [3], [14], [15], [19]. However, there have been hints that these signaling pathways, or components thereof, could have functions that are not restricted to migration along a single axis. For example, UNC-40 is involved in A/P migrations of Q neuroblasts [4], [20] and in A/P motor axon dendrite growth [21]. Moreover, we and others [22], [23] have shown that over-expression of UNC-40/DCC in the mechanosensory neurons causes A/P polarity reversals in ALM and PLM axons akin to the effects of impairing Wnt signaling in these neurons [14], [15], [24]. Intrigued by the possibility of integration between Netrin and Wnt signaling, we examined the effects of simultaneously impairing Netrin and Wnt functions on cells and growth cones that navigate along the A/P, the D/V, or both axes. This revealed previously unrecognized and unexpected, redundant roles for Wnt signaling in D/V guidance, and for UNC-6/Netrin signaling in A/P guidance as well as redundant roles that affect organ function and embryonic viability. We further found that a balance between signaling by UNC-5 and LIN-44/Wnt and between specific Wnts, like EGL-20 and CWN-1, contributes to the regulation of A/P polarity and that in the absence of UNC-6/Netrin function, Wntless and SFRP, and by implication one or more Wnts, are required for a long-sought mechanism that functions in parallel to UNC-6/Netrin signaling to regulate D/V migrations. These findings open new avenues for deciphering how A/P and D/V guidance signals are integrated to establish polarity in multiple biological processes and implicate broader roles for Netrin and Wnt signaling - roles that are hidden due to prevalent redundancy between the functions of these cues.

## Results

### Netrin and Wnt signaling function redundantly to regulate D/V and A/P guidance of migrating Distal Tip Cells (DTCs)

**Figure 1:**
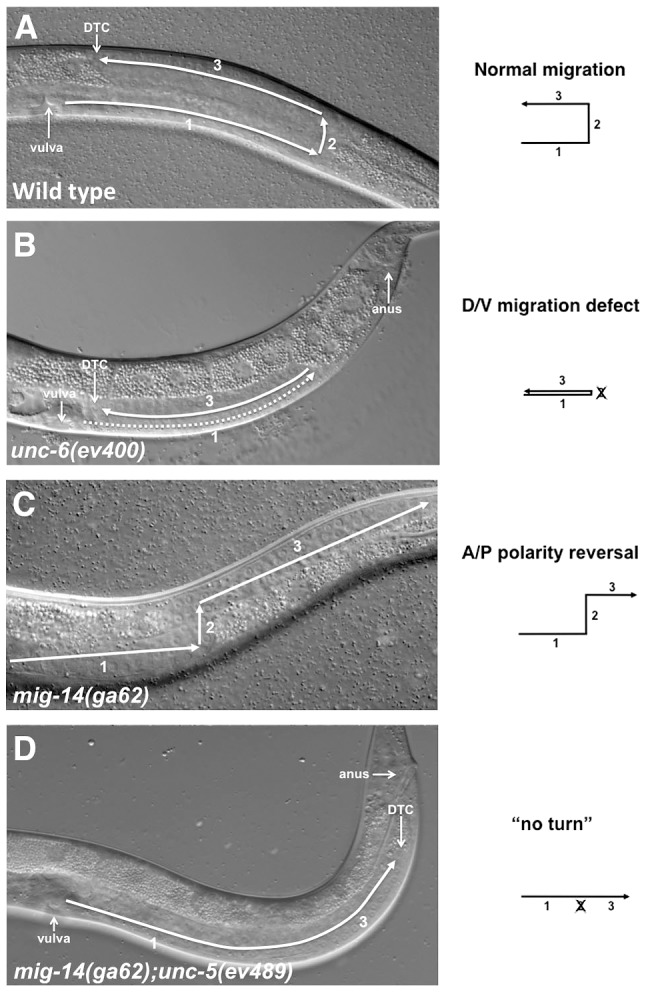
Netrin signaling components and Wntless are involved in guiding DTC migrations. DIC images show migrations of the posterior DTC in L4 stage hermaphrodites. In all panels anterior is left and dorsal is up. (A) In the wild type a posterior U-shaped gonad arm is formed by 3 sequential migratory phases of the posterior DTC. The anterior DTC (not shown) undergoes a mirror image symmetrical pattern of migration. (B) In *unc-6(ev400)* the posterior DTC frequently fails to execute the phase 2 ventral to dorsal migration resulting in a ‘ventralized’ gonad arm. The anterior DTC of these mutants (not shown) also exhibits these phase 2 failures, but at a lower frequency than the posterior DTC. The dashed line represents the gonad segment formed in phase 1 that overlaps the segment formed in phase 3. (C) In *mig-14(ga62)* the first and second phases of migration are normal, but at the onset of phase 3 the DTC frequently displays a polarity reversal and migrates away from the mid-body instead of towards it. In some cases the DTC initially turns towards the mid-body and subsequently reverses its polarity (Figure S1). (D) In *mig-14(ga62); unc-5(ev489)* animals, the posterior DTC frequently fails to execute phase 2 migration; this together with subsequent phase 3 polarity reversals cause a ‘no turn’ phenotype. The diagrams depict the migratory pattern of the posterior DTCs corresponding to the phenotypes shown in the DIC images.

In *C. elegans* hermaphrodites, migration of the DTCs of the somatic gonad represent an excellent model system to study how polarity information provided by extracellular cues is utilized to enact normal migration patterns. The two DTCs are born in the ventral mid-body of the animal and migrate post-embryonically in three sequential phases alternating between the A/P and D/V axes of the body wall as they lead the elongation of anterior and posterior mirror image U-shaped hermaphrodite gonad arms (posterior arm shown in Figure 1A). In phase 1 the anterior and posterior DTCs migrate away from one another along the ventral body wall muscles towards the head and tail, respectively. In phase 2 the DTCs reorient 90° and migrate along the D/V axis of the lateral epidermis. In phase 3 the DTCs reorient again 90° and migrate on the dorsal body wall muscles back to the mid-body of the animal [5].

Many of the genes that regulate DTC migrations, such as Netrins, Wnts, integrins and matrix metalloproteases, are highly conserved and function to guide cell and axon migration in vertebrates and invertebrates [25]. UNC-6/Netrin, through its transmembrane receptors UNC-40/DCC and UNC-5, guide the D/V migrations of the DTCs [5]. In *unc-5*, *unc-6* and *unc-40* loss of function (lof) mutants, the DTCs execute phases 1 and 3 with normal timing but frequently fail to execute phase 2 migration, which is normally mediated by UNC-40 and UNC-5 eliciting migration away from ventral UNC-6 sources [5]. Phase 2 failures cause ‘ventralized’ gonad arms that lie solely over the ventral muscle bands (Figure 1B). The incomplete penetrance of this defect in null mutants of Netrin signaling components (which is also observed in Netrin-dependent axon guidance) suggests the existence of a previously unknown, long-sought signaling pathway that functions in parallel with Netrin signaling to execute D/V migrations.

**Figure 2:**
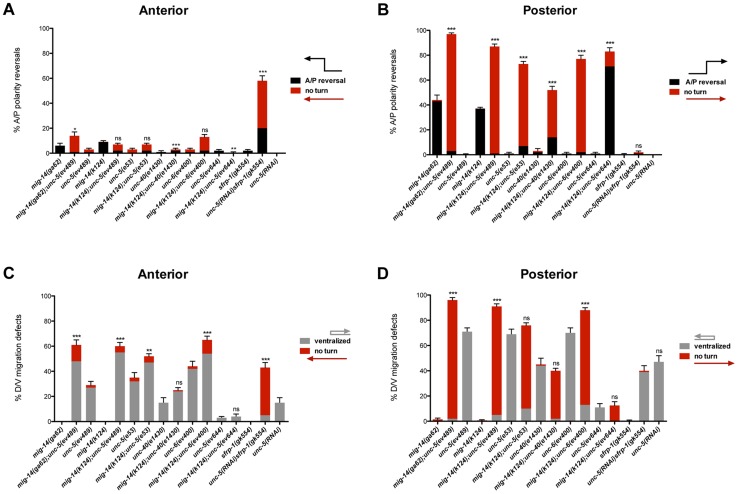
Netrin signaling and *mig-14/wntless* have redundant roles in A/P and D/V guidance of the DTCs. (A, B) Quantification of phase 3 A/P polarity reversals in single mutants of *mig-14/wntless*, *unc-5*, *unc-40*, *unc-6* and *sfrp-1* versus double mutants of *mig-14* with *unc-5*, *unc-40*, or *unc-6* and of *sfrp-1* with *unc-5(RNAi)*. Bars represent the percentage of phase 3 polarity reversals (black) and ‘no turn’ (red) phenotypes. The latter represent a subset of the phase 3 reversals. The corresponding raw data for all panels are presented in Table S2. All strains were analyzed in the background of *dnIs31[gly-18p::gfp]* to visualize the DTCs. *mig-14(ga62); unc-5(ev489)* was also analyzed without *gly-18p::gfp*, which made no difference (Table S2). Most of the progeny of the *mig-14(k124); unc-6(ev400)* double mutant were not viable, therefore, escapers were analyzed. Error bars indicate standard error of the sample proportion. Comparisons of the total phase 3 A/P reversals for double deficits were made to *mig-14(ga62)*, *mig-14(k124)*, or *sfrp-1(gk554)*. ***P<0.00001 **P<0.001; *P<0.05; ns = not significant (P≥0.05). (C, D) Quantification of the phase 2 D/V migration failures in the strains presented in panels (A) and (B), respectively. Bars represent the percentage of ‘ventralized’ (grey) and ‘no turn’ (red) gonad arms. The latter represent a subset of the phase 2 migration failures. The corresponding raw data for all panels are presented in Table S2. Error bars indicate standard error of the sample proportion. Comparisons of the total phase 2 migration failures (‘no turn’ plus ‘ventralized’ gonad arms) were made between paired single and double deficits. ***P<0.00001 **P<0.001; *P<0.05; ns = not significant.

*mig-14* encodes the *C. elegans* homolog of Wntless, a seven transmembrane domain protein necessary for Wnt secretion [17]. When the function of *mig-14* is impaired, phase 2 migration of both DTCs is essentially normal, but phase 3 migrations display a 180° polarity reversal and are frequently mis-oriented away from mid-body rather than towards it (Figure 1C) [26]. We refer to this defect as a phase 3 A/P polarity reversal. *unc-6, unc-5* and *unc-40* lof mutants rarely display phase 3 polarity reversals; however, these reversals are observed when UNC-5 is over-expressed in the DTCs (N. Levy-Strumpf & J. Culotti, in preparation). This prompted us to examine the outcome of simultaneously impairing the function of MIG-14/Wntless and UNC-6/Netrin signaling components. We therefore generated double mutants carrying different combinations of *mig-14/wntless*, with *unc-5*, *unc-40* and *unc-6* alleles (detailed allelic description is provided in Table S1). The DTC migrations observed in these double mutants exhibited one of four migratory patterns: a normal migratory pattern (Figure 1A), a phase 2 D/V migration failure (Figure 1B; Figure 2C, 2D grey bars), a phase 3 polarity reversal (Figure 1C; Figure 2A, 2B black bars), or a combination of a phase 2 failure followed by a phase 3 A/P polarity reversal resulting in a ‘no turn’ phenotype (Figure 1D; Figure 2 red bars).

While neither allele of *mig-14/wntless* caused significant phase 2 D/V migration failures (<1%, n = 1147), both alleles significantly enhanced phase 2 failures caused by *unc-5*, *unc-6* and *unc-40* null mutations. Anterior (Figure 2C; Table S2) and posterior DTCs were markedly affected, with as many as 98% of posterior DTCs exhibiting phase 2 D/V migration failures in the double mutants (Figure 2D; Table S2). A reciprocal situation was found for the regulation of A/P guidance by MIG-14 and Netrin signaling components. While Netrin signaling mutants rarely caused phase 3 polarity reversals, they significantly enhanced the phase 3 A/P polarity reversals of posterior DTCs in *mig-14/Wntless* mutant animals, with some allelic combinations exhibiting almost complete penetrance of the defect (97%, n = 151) (Figure 2B; Table S2). These results reveal redundant roles for UNC-6/Netrin signaling components and MIG-14/Wntless in determining the A/P polarity of the DTC during their phase 3 migration, and together with the previous results demonstrate that the mechanism that functions in parallel with UNC-6/Netrin signaling to regulate D/V DTC migration depends on MIG-14/Wntless, and by implication, on one or more Wnt signals that function redundantly with UNC-6.

Wntless displays greater effect on the posterior DTC as evidenced by the higher frequency of phase 3 A/P polarity reversals of posterior DTCs in *mig-14/wntless* mutants. To further explore a role for Wnts and Netrins in guiding the anterior DTC, we included an additional Wnt regulator in this analysis. *sfrp-1* encodes the *C. elegans* homolog of SFRPs. *sfrp-1* is expressed anteriorly in *C. elegans* and functions to inhibit anterior Wnts such as CWN-1 and CWN-2 [27]. *unc-5* and *sfrp-1(gk554)* mutants displayed a low incidence of A/P polarity reversals (2% n = 230 in *sfrp-1*); nevertheless, simultaneous loss of *sfrp-1* and *unc-5* caused polarity reversals in 58% (n = 189) of the anterior DTCs (Figure 2A; Table S2) supporting the finding of redundant functions for Wntless and UNC-5 signaling in guiding the phase 3 A/P migration of the DTCs. Similar to *mig-14*/*wntless* mutations, the *sfrp-1* mutation also enhanced the D/V guidance defects of *unc-5* lof mutations (Figure 2C).

### The Wnt-Netrin interaction is independent of the dorsoventral position of the DTC

*unc-5* null mutants display a high percentage of posterior DTC phase 2 D/V migration failures resulting in DTCs that remain on the ventral side throughout their migration. This raises the possibility that the *mig-14; unc-5* double mutant enhancement of A/P polarity reversals might be an indirect consequence of the ventral positioning of the DTC. To exclude this possibility, we repeated the *mig-14; unc-5* double mutant analysis using the weak *unc-5(ev644)* allele, which manifests only a low penetrance of phase 2 migration failures [28]. Similar to what we observed with the *unc-5* null mutations, the frequency of *mig-14/wntless* posterior DTC phase 3 A/P polarity reversals was enhanced in the *mig-14(k124); unc-5(ev644)* double from 37% to 83%, [of which 71% occurred on the dorsal side (Figure 2B black bar)]. These results demonstrate that a simultaneous reduction in Wntless and UNC-6/Netrin signaling components causes an increase in A/P polarity reversals regardless of whether phase 3 occurs on the ventral or dorsal side and regardless of whether phase 3 is preceded by a normal or a failed phase 2 migration. Furthermore, the finding that the *mig-14/wntless; unc-5(ev644)* double mutant displays a low frequency of D/V migration defects, but a high frequency of A/P reversals (the same as the *unc-5* null), raises the possibility that the functional requirements for UNC-5 in D/V versus A/P guidance are genetically separable.

### UNC-5 functions redundantly with some Wnt ligands, while opposing the function of others, to regulate phase 3 A/P migration

**Figure 3:**
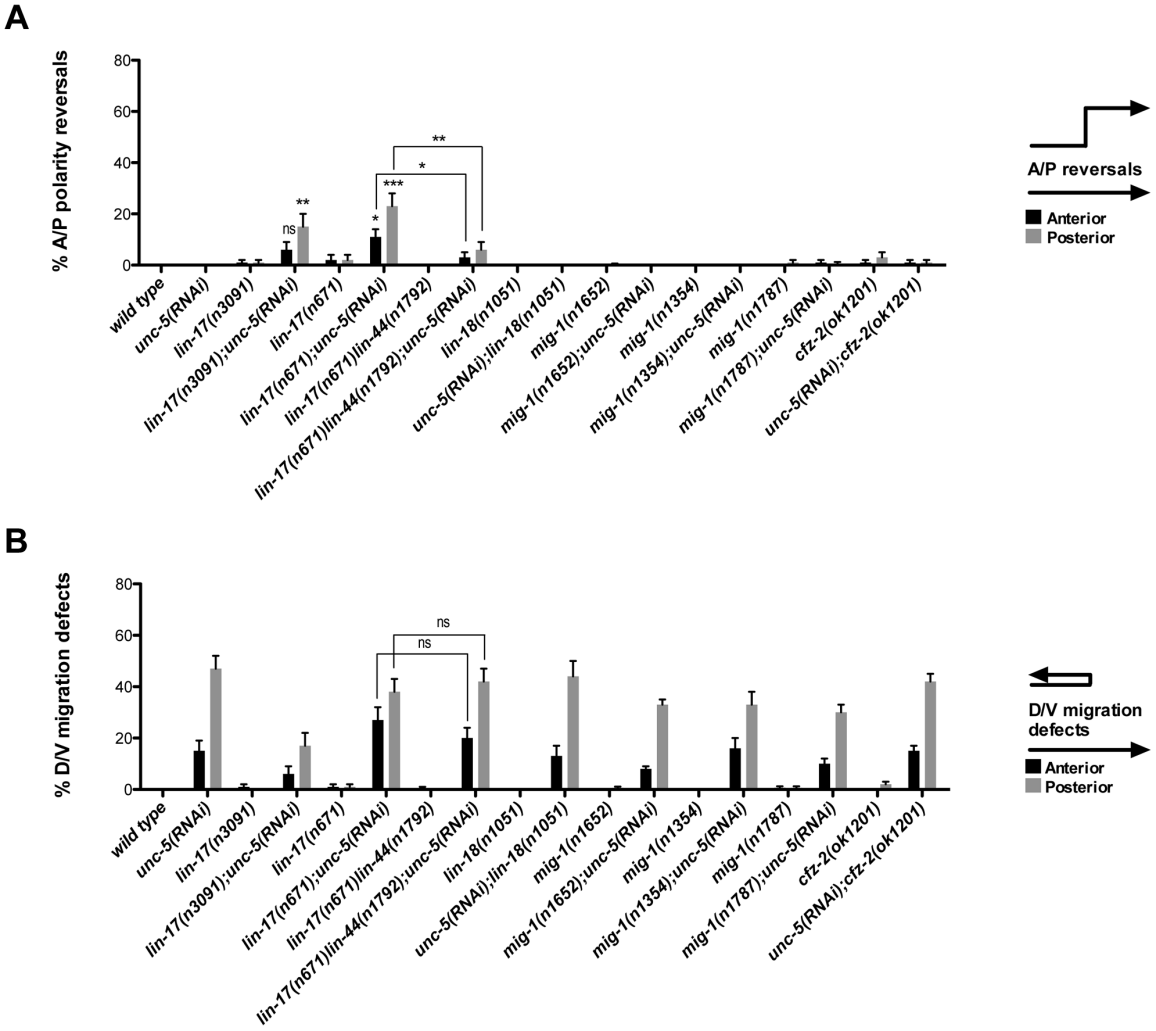
UNC-5 and the Wnt Frizzled receptor LIN-17 function redundantly to determine DTC phase 3 A/P polarity. (A) The effects of *unc-5(RNAi)* on the frequency of phase 3 A/P polarity reversals in single and double mutants of Wnt receptor-encoding genes are shown for anterior (black bars) and posterior (grey bars) DTCs. The corresponding raw data are presented in Table S3. Error bars indicate the standard error of the sample proportion. Comparisons of A/P polarity reversals were made between paired single and double deficits or as indicated by the connecting lines. ***P<0.00001 **P<0.001; *P<0.05; ns = not significant. (B) Quantification of percent phase 2 D/V migration failures in the populations analyzed and presented in (A). The phase 2 D/V migration failures result from impairing *unc-5* function, and reflect the efficacy of the *unc-5(RNAi)*.

To further examine whether the genetic interactions between Netrin signaling mutants and *mig-14/wntless* or *sfrp-1* reflect Wnt signaling defects, we analyzed genetic interactions between *unc-5* and various Wnt- and Wnt receptor-encoding genes. For most of this analysis we used *unc-5(RNAi)* to impair *unc-5* function. *unc-5(RNAi)* causes DTC phase 2 D/V migration failures (visualized as ‘ventralized’ gonad arms) typical of *unc-5* lof alleles, which were quantified to provide a measure of efficacy of the RNAi treatment on UNC-5 function (Figure 3B; Table S3). The effect of *unc-5(RNAi)* on A/P polarity reversals was comparable to that of *unc-5* lof alleles (Figure S3; Table S4).

**Figure 4:**
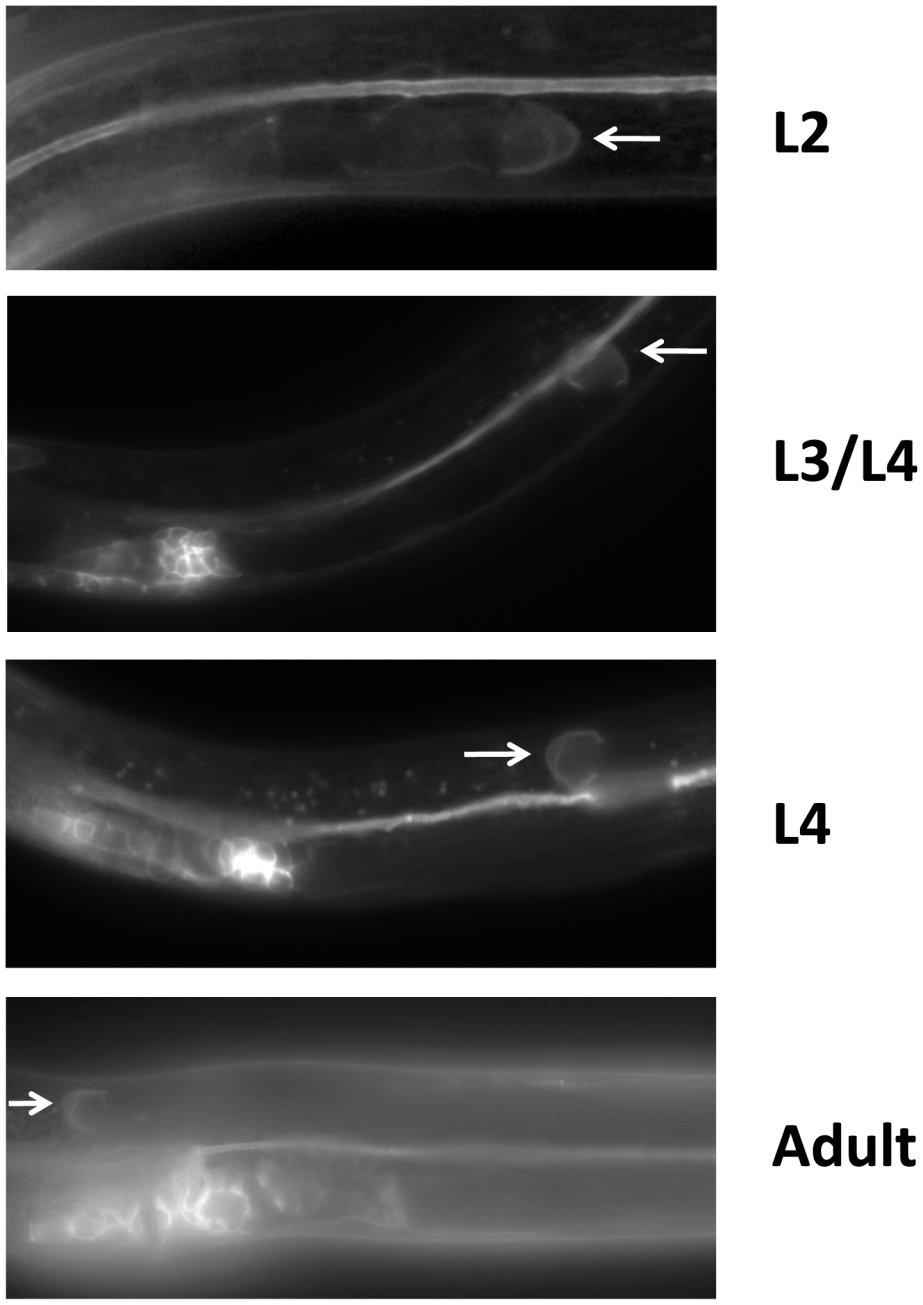
LIN-17 is expressed in the hermaphrodite DTCs throughout development. Fluorescence micrographs of KS411 hermaphrodites bearing *lin-17::gfp* [58]. Dorsal is up and anterior is left. Arrows mark the DTC. Developmental stage is indicated on the right. L2–L4 represent the three larval stages preceding the adult stage.

**Figure 5:**
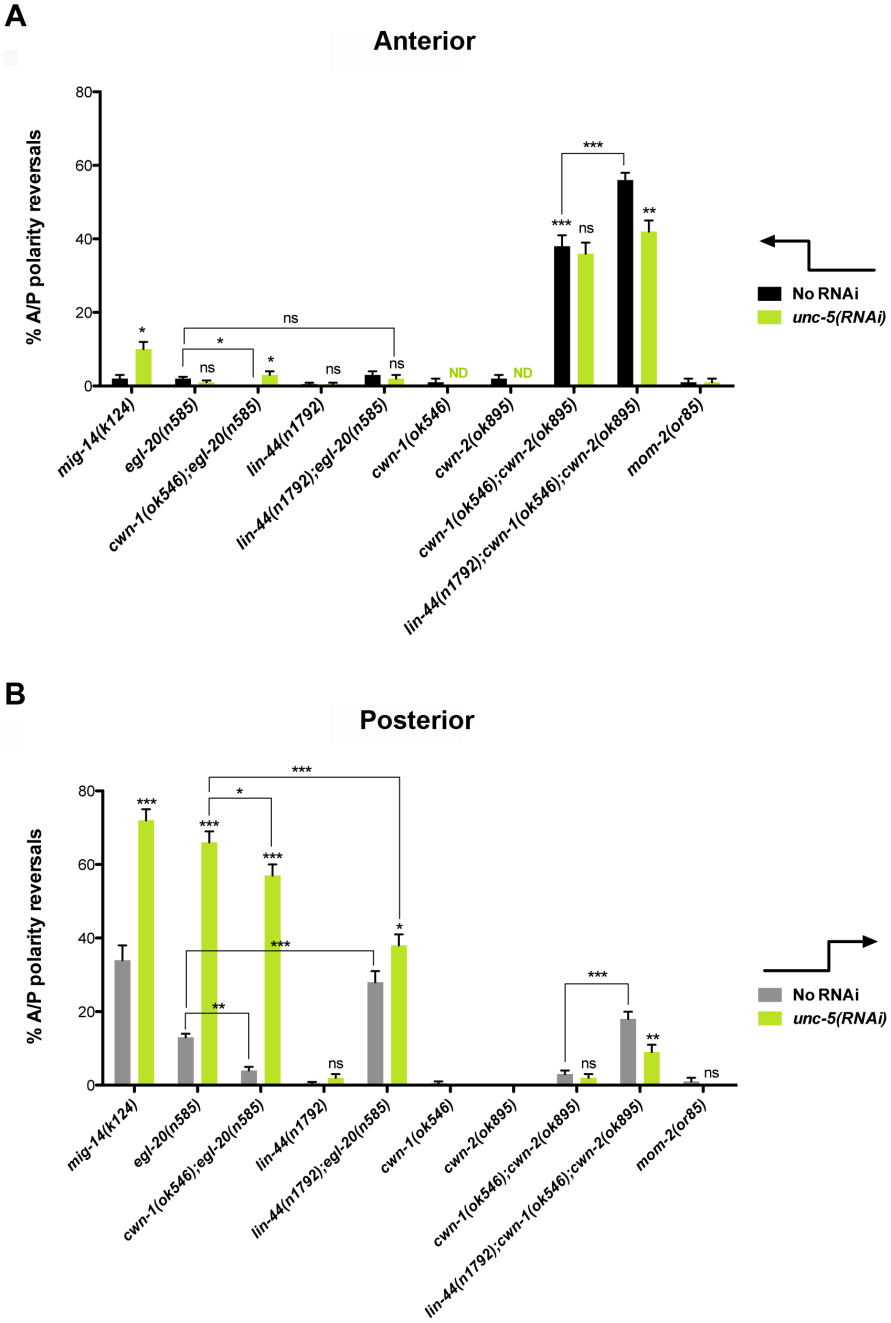
UNC-5 and Wnt ligands function redundantly or in mutual opposition to determine A/P polarity. (A) Shown is the frequency of anterior DTCs exhibiting phase 3 A/P polarity reversals in *wnt* mutants not treated (black bars) or treated (green bars) with *unc-5* RNAi. (B) Shown is the frequency of posterior DTC phase 3 A/P polarity reversals in Wnt receptor mutants not treated (grey bars) or treated (green bars) with *unc-5* RNAi (green bars). The corresponding raw data are presented in Table S5. Error bars indicate standard error of the sample proportion. Comparisons of A/P polarity reversals in multiple mutant lines were made to the corresponding single and double mutant controls or as indicated by the connecting lines. ***P<0.00001 **P<0.001; *P<0.05; ns = not significant.

We examined whether mutations in each of the five Wnt receptor-encoding genes (*lin-17*, *lin-18*, *cfz-2*, *mig-1 and mom-5*) might function redundantly with *unc-5* to prevent phase 3 polarity reversals. Except for *mom-5* alleles, which cause a high frequency of phase 3 DTC A/P polarity reversals [29] (also the subject of a report by N. Levy-Strumpf & J. Culotti, in preparation), these mutations cause few if any phase 3 reversals. For example, two putative null alleles of *lin-17/frizzled*, *(n3091)* and *(n671)*, caused only 1–2% phase 3 DTC A/P polarity reversals, however both alleles were significantly enhanced for these defects by *unc-5(RNAi)* or *unc-5* mutations (Figure 3A; Table S3; Figure S3; Table S4), whereas *lin-18*, *cfz-2*, and *mig-1* mutations were not enhanced (Figure 3, Table S3). Thus, out of the four Wnt receptor genes examined here (*lin-17*, *lin-18*, *cfz-2*, and *mig-1*), only *lin-17* was found to function redundantly with *unc-5* for phase 3 A/P reversals. Consistent with a role for LIN-17/Frizzled in phase 3 polarity determination, we found that GFP-tagged LIN-17 is expressed in the DTCs throughout development (Figure 4), whereas LIN-18, CFZ-2, and MIG-1 are not reportedly expressed in these cells [15], [30], [31] (see also Figure S2). In examining the role of specific Wnts in DTC migration, we found that single *wnt* gene mutations caused few or no DTC migration defects (Figure 5, Table S5); however, simultaneous impairment of *unc-5* and *egl-20/wnt* (by RNAi or by mutation) caused synergistic enhancement of phase 3 A/P polarity reversals from 13% (n = 555) in *egl-20(n585)* to 66% (n = 353) in *unc-5(RNAi) egl-20(n585)* (Figure 5B; Table S5) animals, to 70% (n = 124) in *unc-5(ev489) egl-20(n585)* animals, and to 55% (n = 210) in *unc-5(e53) egl-20(n585)* animals (Figure S3; Table S4). This demonstrates that *unc-5* functions redundantly with *egl-20/wnt* to direct the posterior DTC back to the mid-body during phase 3 just as *unc-5* functions redundantly with *lin-17/frizzled*, *mig-14/wntless* and *sfrp-1* in this process (Figure 2; Figure 3). Interestingly, the frequency of posterior DTC phase 3 A/P polarity reversals in *lin-17; unc-5(RNAi)* and in *unc-5(RNAi) egl-20(n585)* animals was suppressed by *lin-44(n1792)* from 23% to 6% and 66% to 38%, respectively (Figure 3A; Table S3; Figure 5B; Table S5). These results demonstrate that enhancement of *lin-17/frizzled* and *egl-20/wnt* single mutant A/P polarity defects by impaired *unc-5* function requires LIN-44 activity and suggest that a balance between UNC-5 and LIN-44 activities promotes normal DTC phase 3 A/P polarity.

*unc-5(RNAi)* did not enhance the frequency of *cwn-1(ok546); cwn-2(ok895)* double mutant DTC phase 3 A/P polarity reversals (n>300) (Figure 5; Table S5); however, a redundant role for *lin-44/wnt* in anterior and posterior DTC migrations was uncovered when *cwn-1* and *cwn-2* were severely compromised (n>290) (Figure 5). This function of *lin-44/wnt* is partially dependent on *unc-5* as determined by the suppression caused by *unc-5(RNAi)* of the *lin-44; cwn-1; cwn-2* triple mutant (Figure 5; Table S5). These results demonstrate that enhancement of *cwn-1; cwn-2* double mutant phase 3 A/P polarity reversals by a *lin-44* lof requires UNC-5 activity and provides further evidence that a balance between UNC-5 and LIN-44 activities promotes normal DTC phase 3 A/P polarity.

### Multiple Wnt ligands are involved in establishing DTC polarity on the A/P axis

A balance between various Wnts in determining DTC polarity was also observed. We analyzed single or combination mutants of *egl-20*, *lin-44*, *cwn-1*, and *cwn-2*; in all cases null or severe lof alleles were used (Table S1). This analysis revealed either greater than additive enhancement of the phase 3 A/P polarity reversals (indicating redundancy) or mutual suppression of the defects (indicating the requirement for a balance between gene functions). For example, we found that *cwn-1* functions redundantly with *cwn-2*, while *lin-44* functions redundantly with *egl-20* and with *cwn-1* or *cwn-2* (or both) to regulate DTC phase 3 A/P polarity. Conversely, the *egl-20* phase 3 reversals were markedly, but not completely, suppressed by mutations in *cwn-1* (Figure 5A black bars; Figure 5B grey bars; Table S5). This suppression implies that a balance between EGL-20/Wnt and CWN-1/Wnt is also required to promote normal phase 3 A/P polarity.

Most of the Wnts and their receptors, either alone or in combination, mainly affected posterior DTC migration; the only exceptions were *sfrp-1* and the double knockout of *cwn-1* and *cwn-2*, which mainly affected anterior DTC migration (Figure 5A black bars). This corresponds to the expression pattern of CWN*-2* and SFRP-1, which are mainly expressed in the anterior, compared to the posterior expression of LIN-44, EGL-20 and CWN-1 [27]. The *lin-44; cwn-1; cwn-2* triple mutant reveals a redundant role for *lin-44* and the *cwn* genes in both anterior and posterior DTCs. This is interesting given that *lin-44* is the most posteriorly expressed Wnt in L1 larvae [27]. However, this observation is not unprecedented [32]. It is possible that a more anterior source of LIN-44 [27], [30] accounts for this, or that CWN-1, which is expressed more broadly, somehow facilitates the LIN-44 effect on the anterior DTC.

### Redundant functions for Wnts and Netrin signaling components in axon guidance

**Figure 6:**
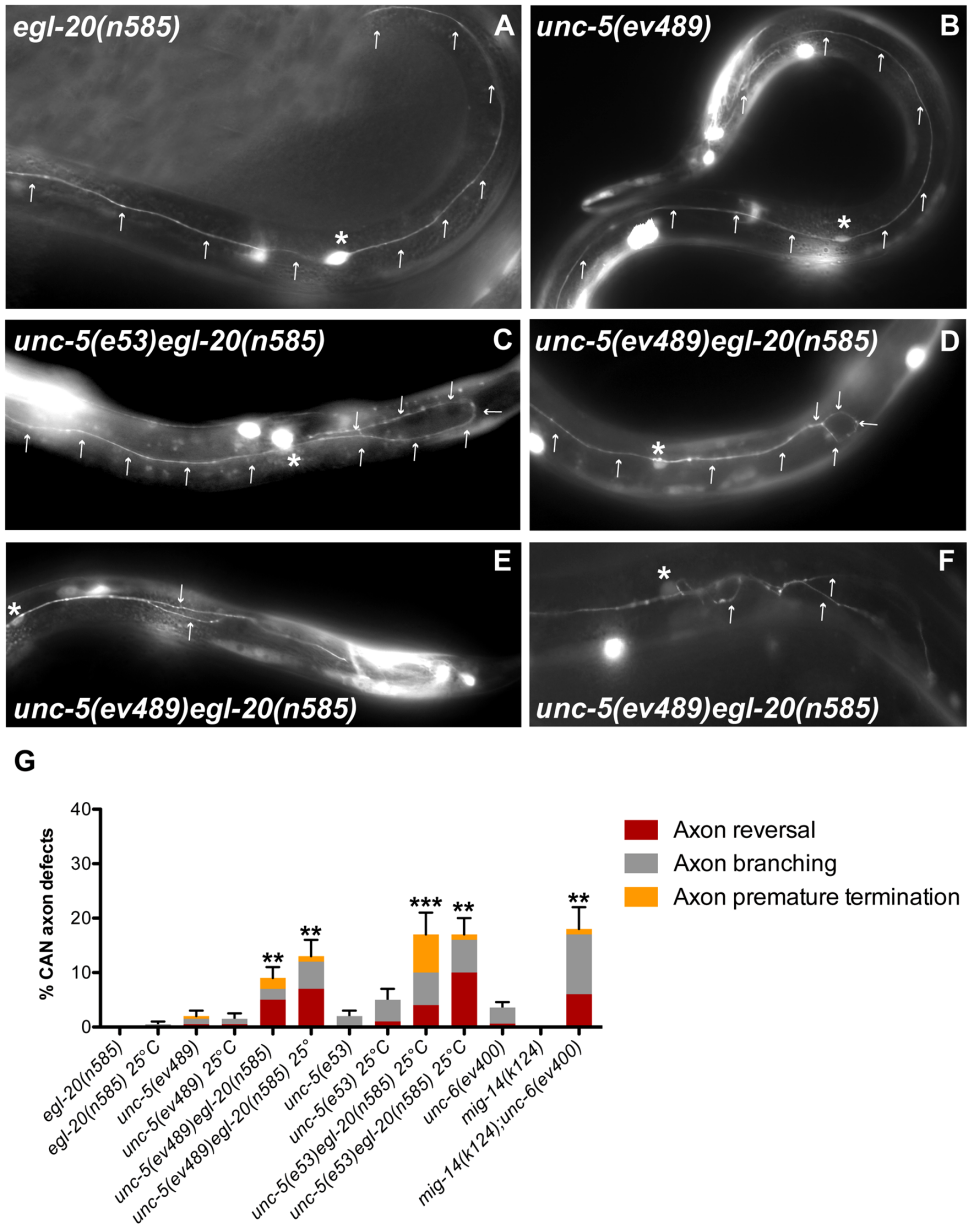
Netrin and Wnt signaling components function redundantly to regulate A/P axon guidance of the CAN neuron. Fluorescence micrographs of the indicated mutant strains bearing *gly-18::gfp* to visualize the CAN neuron. Dorsal is up and anterior is left. Arrows mark the CAN axon trajectories. Asterisk marks the CAN cell body. (A) *egl-20(n585)* and (B) *unc-5(ev489)* both display bipolar axon extensions along the A/P axis as in the wild-type. (C) *unc-5(e53) egl-20(n585)* as well as (D) *unc-5(ev489) egl-20(n585)* display axon polarity reversals, as well as axon branching and premature terminations (E,F) All of these defects occur predominantly on the posterior side. (G) Quantification of the CAN axon defects in *egl-20*, *unc-5*, *mig-14/wntless* and *unc-6* single mutants compared to *unc-5 egl-20* or *mig-14; unc-6* double mutants, respectively. *egl-20(n585)* is reportedly temperature sensitive [33] hence the analysis was carried out at both 20°C and 25°C. Bars represent the percentage of CAN axon reversals (red), branching (grey), and premature terminations (yellow). The corresponding raw data are presented in Table S6. Error bars indicate standard error of the sample proportion. Comparisons were made to the corresponding single mutant controls. ***P<0.00001 **P<0.001.

To examine whether axon pathfinding is also regulated by redundant functions of Wnt and Netrin signaling, we determined whether *unc-5 egl-20*, or *mig-14/wntless; unc-6/netrin* double mutants display any synergistic axon guidance defects. We analyzed two different types of neurons: the CAN neuron, which is bipolar and extends axons along the A/P axis (Figure 6A and 6B), as well as the mechanosensory neurons, which extend axons along the A/P and D/V axes.

Single *unc-5(e53)*, *unc-5(ev489)* or *egl-20(n585)* mutants rarely displayed CAN axon guidance defects (Figure 6G; Table S6), whereas the *unc-5 egl-20* double mutants displayed a synthetic defect resulting in 5–10% (n>100) CAN axon reversals, which were observed predominantly in the posterior axon (Figure 6C, 6D, and 6G). Other defects such as premature axon termination and excessive branching (Figure 6E, 6F) were also observed, resulting in a total of 9–17% defects (Figure 6G) depending on the allele or the incubation temperature [33]. Similarly, *unc-6(ev400)* and *mig-14(k124)* mutants rarely displayed CAN axon guidance defects, while the penetrance of these defects in *mig-14(k124); unc-6(ev400)* double mutants was 18% (n = 104). These results suggest a role for Netrin signaling in guiding CAN axons - a role that is redundant with Wnt signaling, which has established instructive and permissive functions in A/P guidance of several other axons in *C. elegans* [14], [15]. These results are consistent with the apparent Wnt-redundant role of UNC-6/Netrin signaling in A/P guidance of DTCs.

**Figure 7:**
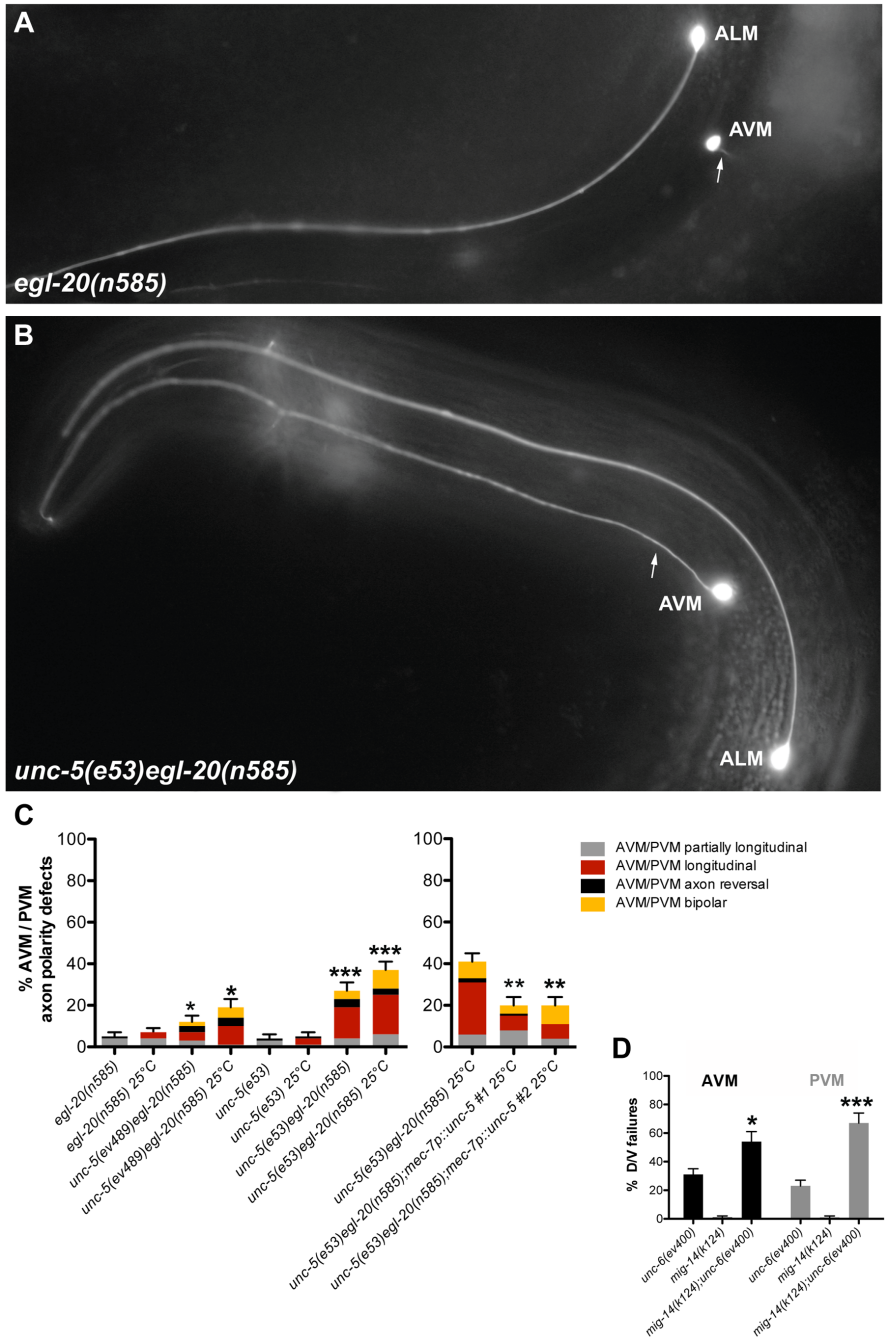
Wnt and Netrin signaling components function redundantly to regulate D/V axon guidance of AVM and PVM neurons. (A) *In egl-20(n585)* mutants, the AVM neuron sends an axon (arrow) ventrally as in the wild type. (B) *unc-5(e53) egl-20(n585)* double mutants frequently fail to execute the normal D/V axon migration and the AVM axon migrates longitudinally instead. (C) Quantification of the AVM or PVM axon defects in *egl-20* and *unc-5* single mutant compared to *unc-5 egl-20* double mutant animals or the *unc-5 egl-20* compared to two independent lines of this double mutant carrying the *mec-7::unc-5* transgene. Bars represent the percentage of longitudinal axons (red) or partially longitudinal axons (grey). A/P polarity defects including axon reversals (black) and bipolar axons (yellow) were also observed. (D) Quantification of the AVM (black) or PVM (grey) axon defects in *mig-14/wntless* or *unc-6* single mutants compared to *mig-14; unc-6* double mutants. The corresponding raw data are presented in Table S7. Error bars indicate standard error of the sample proportion. Comparisons were made to the corresponding single mutant controls. ***P<0.00001; *P<0.05; ns = not significant.

To explore D/V axon guidance, we analyzed the mechanosensory AVM and PVM neurons, which normally extend axons toward ventral sources of UNC-6 [5] (AVM is shown in Figure 7A). A marked enhancement of AVM and PVM axon guidance defects was observed when both Wnt and Netrin signaling were impaired (Figure 7; Table S7). Although *unc-5* and *egl-20* mutants each displayed mild D/V guidance defects (e.g., partially longitudinal axons) and low frequency severe defects (entirely longitudinal axons) mainly at 25°C (Figure 7C; Table S7), the *unc-5 egl-20* double mutants displayed greater than additive penetrance reaching approximately 40% defects at 25°C (n = 125) (Figure 7B, 7C; Table S7). UNC-5 was not known to be involved in attraction towards ventral sources of its ligand UNC-6 [34], but rather to elicit migration away from these sources. These results demonstrate that UNC-5 has a role in guidance toward ventral sources of UNC-6/Netrin that extends beyond its conventional instructive role in axon repulsion [5], [6], [35], [36], and that this role is redundant with a role for EGL-20 in D/V guidance. Observations implying a role for UNC-5 in apparent attraction of HSN axons to ventral sources of UNC-6 were recently made independently by another group [37].

A/P polarity reversals were also observed in the touch neurons of *unc-5 egl-20* double mutants; these included axon reversals of the AVM, PVM or, in rare instances, the ALM axon (Figure 7C; Table S7). Taken together these observations demonstrate that Wnt and Netrin signaling, as they do in phase 2 and phase 3 DTC migration, also function redundantly in regulating D/V and A/P guidance, respectively, of migrating axons (e.g., CAN, AVM, PVM and ALM).

To determine whether the unconventional role of UNC-5 in AVM and PVM axon guidance is cell autonomous, we expressed *unc-5* under the *mec-7* mechanosensory neuron-specific promoter and assayed its ability to rescue AVM and PVM axon guidance defects of the *unc-5(e53) egl-20(n585)* double mutant. Combined defects were rescued by roughly half, whereas the severe AVM and PVM guidance defects were rescued by nearly 60% (Figure 7, Table S7), suggesting that at least part (and perhaps all) of UNC-5 function in this context is cell autonomous.

### Redundancy between Wnt and Netrin signaling in organ function and processes essential for viability

In addition to the redundancy observed for Wnts and UNC-6/Netrin in DTC migration and axon guidance, in the process of generating the *mig-14/wntless; unc-6/netrin* double mutants we observed synergistic effects on vulval morphology and function resulting in a nearly complete egg laying defect (Figure S4A) as well as a higher incidence of protruding vulvae (Figure S4C). A marked synergistic effect was also observed on viability. The *mig-14(ga62); unc-6(ev400)* double mutant could not be generated, while the weaker *mig-14(k124)* allele in combination with the *unc-6(ev400)* null allele displayed extensive embryonic lethality causing extremely low brood sizes averaging less than 10 worms (Figure S4B). Arrested and malformed embryos were frequently observed in the *mig-14(k124); unc-6(ev400)* gonads (Figure S4D and S4E). Although embryonic lethality could be secondary to the egg laying defect, comparing the brood sizes of *unc-6(ev400)* hermaphrodites that failed to lay any eggs to those of *mig-14(k124); unc-6(ev400)* hermaphrodites indicated a greater reduction in viability in the double mutant. These results indicate that Wnts and UNC-6/Netrin function redundantly to orchestrate both vulval function and at least one essential developmental process critical for early development in *C. elegans*.

## Discussion

### Wnts and Netrin signaling components govern D/V and A/P guidance in *C. elegans*

We have identified and characterized unconventional roles for Wnts and UNC-6/netrin in guiding cell and axon growth cone migrations in *C. elegans*. Graded distributions of Netrins and Wnts along the dorso-ventral (D/V) and antero-posterior (A/P) axes of the body wall, respectively, have long been thought to provide polarity information for migrations along these respective axes since UNC-6/Netrin signaling deficits were originally found to primarily affect migrations along the D/V axis and Wnt signaling deficits were found to primarily affect migrations along the A/P axis. Here we have characterized the effects of individual and combined loss of function of Wnt and Netrin signaling components on the migrations of cells (the hermaphrodite DTCs) and axons (of CAN, AVM, PVM and ALM neurons) *in vivo*. Our analysis indicates that the idea that guidance cues like Netrins and Wnts contribute to guidance only along the axis of their gradation is an oversimplification. We found that compromising Wnt signaling reveals an unexpected, redundant role for Netrin signaling components in orienting DTC migration along the A/P axis and, conversely, compromising Netrin signaling reveals an unexpected, redundant role for Wnt signaling in guiding DTC migration along the D/V axis. These findings indicate that Netrins and Wnts have two major functions in guiding migrations. One is to provide instructive polarity information along the axis of their gradation and the other is to help guide migrations orthogonal to the axis of their gradation by functioning redundantly with each other. These results demonstrate that Wnt signaling can function independently of, but redundantly with, Netrin signaling to promote D/V oriented DTC migration and that UNC-5 signaling can function independently of, but redundantly with, Wnt signaling to promote A/P oriented DTC migration. Furthermore, our finding that the *unc-5(ev644)* hypomorphic allele has impaired A/P guidance, but almost intact D/V guidance, indicates that the unconventional function of UNC-6, UNC-5, and UNC-40 in DTC phase 3 A/P polarity may be genetically separable at the level of UNC-5 function from conventional instructive UNC-6 signaling [36]. These results provide a conceptually novel view of how A/P and D/V guidance mechanisms can be regulated *in vivo*.

Notably, simultaneous compromise of both Wnt and Netrin signaling pathways caused the nearly complete penetrance of posterior DTC phase 2 D/V and phase 3 A/P migration defects, demonstrating that the sum of Wntless and Netrin signaling accounts for the entire phase 2 and phase 3 guided migrations of this cell. This together with the markedly increased frequency of AVM or PVM D/V guidance defects in the *mig-14; unc-6* double mutants, compared to the *unc-6* nulls, identifies Wnt signaling as the long-sought mechanism postulated to function in parallel to Netrin in regulating D/V guidance in migrating cells and axons in *C. elegans* and possibly across different species.

The phenotypes observed in the *mig-14; unc-5* and *mig-14; unc-6* double mutant are reminiscent of defects observed in *src-1* mutants. Furthermore, *src-1* mutant defects are suppressed by loss-of-function mutations in Rho family GTPases [38]. This suggests the possibility that both the Netrin and the Wnt signaling pathways are either regulated by SRC-1 or converge on SRC-1 to regulate small GTPase activity known to be critical for A/P polarity establishment [39], [40]. SRC-1 also binds integrins, which are involved in the regulation of the phase 3 turn [25], [41]. It would be interesting to explore further how these three signaling pathways (Wnts, Netrin, and integrins) are integrated to regulate the turning of the DTC, which must involve coordination of the cytoskeletal rearrangements and adhesion processes.

Netrin signaling components are known to have a greater impact on the migration of posterior DTCs [5]. The data presented here reveal a more comprehensive contribution of the Netrin guidance system to D/V migration of the anterior DTC that is evidently masked by Wnt redundant functions. We speculate that anterior, like posterior DTC guidance, is likely to be fully governed by Netrin and Wnts. Although anterior Wnts, like the CWNs and the anterior Wnt regulator SFRP-1, have greater effects on anterior DTC migration, these effects are not fully penetrant even when a Netrin signaling component is simultaneously impaired. Which of the Wnts other than the CWNs govern anterior DTC migrations remains to be determined. The difference in response of the anterior and posterior DTCs seems to be dependent on the composition of Wnts graded oppositely along the A/P axis. This difference in Wnt responsiveness is likely necessary to facilitate the mirror image migration of the anterior and posterior DTCs.

### A balance of Wnt and UNC-5 signals regulates phase 3 DTC polarity

**Figure 8:**
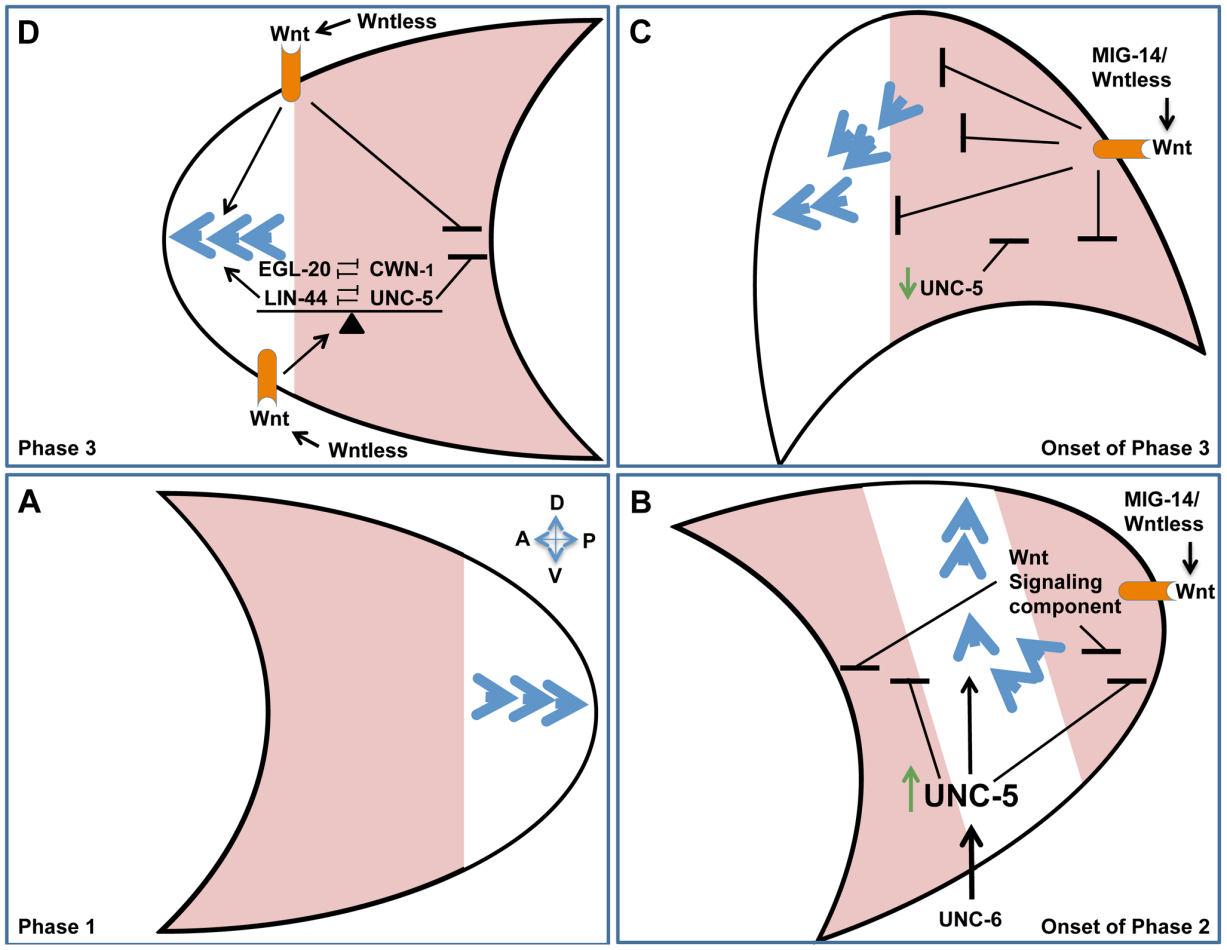
Orchestrating A/P and D/V guidance: A model. Polarity establishment of the posterior DTC (crescent shape) is used as an example to illustrate the redundant functions of Netrins and Wnts in A/P and D/V guidance. Anterior is to the left and dorsal is up. Blue arrowheads represent cytoskeletal polarity establishment regulators. The arrowheads points toward the leading edge of migration. During phase 1 of DTC migration, polarity is established along the A/P axis, away from the mid-body of the animal. (A) The posterior DTC migrates posteriorly (as a result of polarity establishment at the posterior pole) while the anterior DTC (not shown) migrates anteriorly. Areas shaded in pink represent regions from which polarity establishment is inhibited or excluded. (B) At the onset of phase 2, *unc-5* is transcriptionally activated (green arrow) and functions redundantly with Wnt signaling components (Wnt receptor shown in orange) to inhibit polarity formation at the anterior and posterior poles, thereby restricting functional rate limiting cytoskeletal polarity regulators (blue arrowheads) to the center of the cell, confining them to the D/V axis by virtue of excluding them from the A/P poles. In addition to inhibiting A/P polarities, UNC-5 establishes directionality along the D/V axis by inducing repulsion away from ventral sources of UNC-6. The concomitant inhibition of A/P polarity and induction of D/V polarity may be essential for a sharp 90° turn taken by the migrating DTC as it transitions from the A/P to the D/V axis. (C) At the onset of phase 3, UNC-5 appears to be down-regulated [36] and the DTC is located further away from the ventral Netrin source, hence D/V polarity may no longer be maintained and the balance between UNC-5 and Wnt signaling determines polarity establishment asymmetrically on the anterior pole of the posterior DTC, thereby facilitating migration back towards the mid-body. (D) Phase 3 polarity maintenance is dependent on the balance (indicated by a triangular fulcrum) between Wnt and UNC-5 signaling. This balance determines on which of the poles, if any, polarity can be established. There are therefore three possible scenarios: 1) limiting polarity to the posterior or 2) limiting it to the anterior pole, which would result in posterior or anterior migration, respectively, or 3) Limiting polarity to the center of the cell, which would result in a halt, or a migratory transition to the D/V axis. Any of the scenarios (or some combination) could be regulated by the balance between Wnt and UNC-5 signaling.

We identified redundant roles for Wntless or Wnt signaling components and UNC-5, while in a variety of compromised genetic backgrounds we observed mutual suppression of *lin-44* and *unc-5* mutant phase-3 A/P polarity defects. These findings indicate that UNC-5 cooperates with some Wnts and opposes the function of other Wnts to maintain a fine balance of activities required for proper A/P polarity. Similar interactions occur between the different Wnts. Various combinations of Wnt double mutants resulted in synthetic enhancement of phase 3 A/P polarity reversals revealing redundancies between different Wnt signaling components, while on the other hand EGL-20 and CWN-1 display opposing functions. A fine balance of Wnt signaling was similarly reported to be required for migration and positioning of other cell types in *C. elegans* [14], [24], [32], [42]. Our results reveal a contribution of UNC-5 to this balance and imply the existence of a complex regulatory network of interactions between the different Wnts and UNC-5 that determines the phase 3 A/P polarity of DTC migration (Figure 8D; diagrammed in Figure S5). We propose that the balance between Wnts and UNC-5 determines whether anterior or posterior polarities are established (and hence the direction taken on the A/P axis), whether the cell halts, or whether the cell reorients to the D/V axis. The ability of UNC-5 to oppose some Wnts can also explain in principle how an *sfrp-1* lof mutation, which is predicted to up-regulate interacting Wnts, is able to enhance rather than suppress *unc-5(RNAi)*-induced DTC migration defects.

### Orchestrating A/P and D/V guidance: A model

Our data demonstrate that UNC-6 signaling has a role in A/P guidance that is redundant with Wnt mediated A/P guidance, while at the same time Wnts have a role in D/V guidance that is redundant with UNC-6 and UNC-5 mediated D/V guidance. These observations raise a perplexing question: How can Wnt and UNC-6/Netrin signals contribute to D/V and A/P guidance, respectively, when they appear to be graded along orthogonal axes (i.e., A/P and D/V axes, respectively) of the body wall? For the Netrin-redundant contribution of Wnts to D/V guidance, we propose that one or more A/P graded Wnts inhibits the possibility of leading edge formation at the anterior and posterior ends of the cell or growth cone, thereby facilitating D/V guidance - possibly by inhibiting or excluding the polarity establishment machinery from the A/P poles and effectively localizing it to the center of the cell (along the D/V axis) where is can be employed for D/V guidance (Figure 8). This alone would not provide polarity information for the ensuing phase 2 ventral to dorsal migration; however, a possibly analogous situation is provided by early gastrulation in the *C. elegans* embryo. In this case, ingression of endodermal precursors is regulated by a Wnt-frizzled signaling pathway that induces (what could be considered a ventral to dorsal) cell movement by activating apically localized myosin II contraction, thereby effectively squeezing the cell into the embryo's interior [43]. Polarity information in this case may be provided by an intrinsic polarity of the moving cells that is simply activated by Wnt signaling. We propose that this may be akin to the unconventional regulation of phase 2 DTC migration by Wnt signaling. Ventral to dorsal migration is further driven by the conventional instructive ability of an UNC-6/netrin gradient to mediate apparent repulsion by signaling through an UNC-5-dependent receptor mechanism ([36] and see below).

It is worth noting that an *unc-5* mutation behaves like a Wnt signaling mutation in its effects on A/P polarity. Therefore, whatever functions we attribute to Wnt signaling may also be attributed to UNC-5 signaling. This raises the possibility that unconventional UNC-5 signaling contributes to ventralward AVM axon guidance (and perhaps also DTC D/V guidance) by inhibiting anterior and posterior leading edges as proposed above for Wnts. This role of UNC-5 is strictly redundant with the role of EGL-20/Wnt and is separate from UNC-5's instructive ability to mediate repulsion away from ventral sources of UNC-6. An example of apparent A/P bipolar inhibition was recently published in a study of HSN axon guidance, where it was observed that Wnt signaling components function to exclude UNC-40/DCC localization from the anterior and posterior poles of the HSN growth cone [37]. This report lends additional support to the mechanistic specifics of our model.

In a minor variation of the bipolar inhibition model, differential inhibitory effects of UNC-5 on anterior and posterior poles may determine the A/P polarity of axon and DTC phase 3 migrations by skewing the balance between UNC-5 and Wnt inhibitory signals toward one pole. It is tempting to speculate that bipolar inhibition of anterior and posterior leading edges could also serve as a general mechanism for regulating cessation of cell migration along a single axis as well as reorientations from one axis to another, thus determining either cell positioning along the A/P or D/V axes at the end of a migratory path, or changes in trajectory from one axis to another in complex pathfinding processes. The dual function of UNC-5 in A/P and D/V guidance also raises the untested possibility that both Wnt and Netrin signaling pathways converge on the UNC-5 receptor and that this receptor assimilates information from both cues.

### Wnts and Netrins - more than meets the eye

The role of Netrin and its receptors is not limited to cell and axon guidance - it contributes to a wide range of biological processes, including organogenesis, synaptogenesis, dendritic self-avoidance, cell adhesion, angiogenesis, cell survival, tumor formation and metastasis [44]–[48]. Like Netrins, Wnts control a variety of developmental processes including cell migration and axon guidance, synaptogenesis, polarity establishment, cell fate determination, mitotic spindle reorientation, and are also involved in tumorigenesis and various human diseases [4], [10], [13], [49]–[51]. Here we show that Wnts and Netrin signaling components share redundant functions, which are not readily revealed except by impairing both pathways simultaneously, suggesting that they might be substantially involved in more processes and to a greater extent than currently appreciated. The finding of shared functions suggests that Wnt and Netrin signaling mechanisms could be co-regulated. One putative co-regulator is the DAF-12 steroid hormone receptor, which is required for all DTC reorientations [52], [53]. DAF-12 is responsible for the transcriptional activation of UNC-5, just prior to the reorientation of the DTC from the A/P to the D/V axis [36], which in turn helps drive UNC-6 dependent ventral to dorsal phase 2 DTC migration. The nearly full penetrance of the *mig-14; unc-5* double for phase 2 and phase 3 DTC defects raises the distinct possibility that one or more Wnt signal transduction components is co-regulated by DAF-12 along with the UNC-5 receptor not only at the first, but possibly also at the second turn of the DTCs.

Interestingly, SFRPs, known to function as Wnt regulators, contain a cysteine rich domain (CRD), which is highly homologous to the Frizzled family CRD, but also contain a Netrin-related motif (NTR domain) [54]. It is an interesting possibility that the SFRPs may function in some cases to co-regulate these two fundamental pathways.

Redundancy between Wnt and Netrin signaling components extends beyond DTC and growth cone migration to include functions essential for viability. We have observed that double mutants of *unc-6(ev400)* with *mig-14(ga62)* are inviable. These results suggest that certain Wnts and UNC-6 may also function redundantly to regulate at least one essential developmental process critical for viability. Furthermore, the *mig-14; unc-6* double mutants displayed a dysfunctional vulva and a fully penetrant egg-laying defect. Given the involvement of Netrin and integrins in anchor cell invasion [44], which is reminiscent of their involvement in DTC phase 3 A/P polarity [25], it is possible that the egg laying defect is the result of redundancy between Wnt and Netrin function in regulating anchor cell invasion. Taken together, our observations imply that during normal development as well as in some pathological conditions, Wnts and Netrins may have functions that are not apparent due to their redundant output - a notion that is important to consider in order to fully elucidate the underlying mechanisms governing these processes. Furthermore, the observation that Netrins and Wnts have shared functions in A/P and D/V guidance is an important potential precursor to understanding how polarity information from these two guidance systems is integrated to generate defined migratory patterns. Our data provides a novel conceptual view by which D/V and A/P polarity establishment may be effectively one in the same, D/V polarity being, in part, the culminating result of bipolar inhibition of polarity along the AP axis.

## Materials and Methods

### Nematode culture

Standard procedures were used for the culture, maintenance and genetic analysis of *C. elegans* [55]. All strains were grown at 20°C for analysis, unless indicated otherwise. Mutant strains and transgenic lines used in this study are listed in Table S1. Strains not isolated in our laboratory were obtained from the *Caenorhabditis elegans* Genetics Center (University of Minnesota), or as indicated in the Acknowledgements section. When necessary, double mutants were verified by PCR; primers are listed in Table S1.

### RNA interference (RNAi)

*unc-5(RNAi)* constructs were generated by cloning a 574 bp *EcoRI fragment* spanning nucleotides 563–1137 of *unc-5* into the pPD129.36 L4440 vector [56]. In vitro transcribed RNA (Ambion MEGAscript kit) was then injected into young adult hermaphrodites by standard procedures [57]. F1 progeny of the injected worms were analyzed as L4 larvae or adults and compared to the respective non-injected strains.

### Microscopy

DTC migration patterns or axon pathfinding were scored by mounting 1 mM levamisole-treated animals (L4 or adult stage) on 2% agarose pads for observation using Differential Interference Contrast (DIC) and fluorescence microscopy (Leica DMRA2 or DMRB microscope). All strains assayed in Figure 2 and described in Table S2 carried the *gly-18p::gfp* transgene to mark the DTCs. *gly-18p::gfp* rarely affects D/V or A/P guidance of the DTC (Table S2). The polarity reversal phenotype is highly dependent on the incubation temperature. Care was taken to analyze all comparable strains under the same growth conditions, therefore, a control strain grown under the same conditions was included in each set of experiments; data from several independently generated lines were analyzed and the data pooled. *gli-18::gfp* was also used to mark the CAN neurons (Figure 6), while *mec-7::gfp* or *mec-4::gfp* were used to label the mechanosensory neurons (Figure 7).

### Lethality and egg laying assays

In order to assess the frequency of the egg laying defects and brood sizes, young hermaphrodites (no older than the L3 larval stage) of *unc-6(ev400)* or *mig-14(k124)* mutants or the *mig-14(k124); unc-6(ev400)* double mutants were cloned and followed over a period of about 5 days. Worms were scored as having an egg-laying defect only if they fully failed to lay eggs. In these cases the parent becomes a “bag” of trapped larvae that eventually eat their way out. To assess brood sizes, the progeny of each cloned worm was counted. It should be noted that the reported brood sizes of the *mig-14(k124); unc-6(ev400)* are an over-estimation of the actual viable propagating progeny, as not all larvae develop fully to the adult stage. Therefore the synergistic effect between these two mutations is likely even greater than presented.

### Statistical analysis

Standard errors of the proportion (SE) were calculated assuming a binomial distribution of the observed proportion and the actual sample size. Statistical tests were carried out using a standard (two-tailed) comparison of two proportions (Z test). All P values represent the probability that the measured frequency of the phenotype is the same for the two strains being compared. A P-value of less than 0.05 is considered significant. All comparisons described as significant in the Results section were based on this criterion.

## Supporting Information

Figure S1 *mig-14/wntless* displays phase 3 polarity reversals. DIC image shows migration of the posterior DTC. Anterior is left and dorsal is up. L4 stage worm is shown. In some *mig-14(ga62)* animals the DTC initially reorients back to the mid-body and only subsequently reverses its polarity 180° to migrate away from the mid-body of the animal.
(TIF)

Figure S2 LIN-18 is not detected in the hermaphrodite DTCs throughout development. DIC and fluorescence micrographs of hermaphrodites carrying *syIs75*, an integrated *lin-18::gfp* transgene array. Anterior is left and dorsal is up. Arrows mark the DTC. Developmental stage is indicated on the right. L2–L4 represent larval stages preceding the adult stage.
(TIF)

Figure S3 *unc-5(RNAi)* phenocopies *unc-5* loss-of-function mutations. The effects of *unc-5(e53)* or *(ev489)* alleles versus *unc-5(RNAi)* on the frequency of phase 3 A/P polarity reversals in *mig-14/wntless*, *egl-20/wnt* or *lin-17/frizzled* mutants are shown as the percentage of phase 3 A/P polarity reversals for anterior (top panel, black bars) or posterior (bottom panel, red bars) DTCs. The corresponding raw data are presented in Table S4. Error bars indicate the standard error of the sample proportion.
(TIF)

Figure S4 Netrin and Wnt signaling components function redundantly to regulate vulval morphogenesis, vulval function and processes essential for viability. In all panels anterior is left and dorsal is up. (A) Bars represent the percentage of egg laying defects in *unc-6(ev400)* or *mig-14(k124)* animals compared to the *mig-14(k124); unc-6(ev400)* double mutant animals. Error bars indicate standard error of the sample proportion. Comparisons were made to the corresponding single mutant controls. ***P<0.00001. (B) Plot of the individual brood sizes of *unc-6(ev400)* and *mig-14(k124)* mutant hermaphrodites compare to *mig-14(k124); unc-6(ev400)* double mutant hermaphrodites. (C–E) DIC images of *mig-14(k124); unc-6(ev400*) hermaphrodites. (C) *mig-14(k124); unc-6(ev400)* double mutants frequently display malformed, protruding vulvae. (D) Arrested, or (E) malformed embryos are frequently observed in the *mig-14(k124); unc-6(ev400*) hermaphrodite gonads.
(TIF)

Figure S5 Summary of genetic interactions observed between mutants of different Wnt signaling components or between Wnt components and UNC-5. The extent of each arrow reports the approximate percentage of phase 3 polarity reversals caused by a Wnt signaling component deficit or an *unc-5* deficit (color coded). Deficits caused by lof mutations are denoted by (-) and those caused by RNA interference are denoted by (RNAi). The summed extent of the grey and red arrows on the same horizontal line pointing in the same direction represents the enhanced penetrance of the multiple mutant deficits, indicating redundant functions. Green arrows pointing in the reverse direction represent the extent of suppression of the above double mutants by an additional deficit (green), indicating opposing functions. The color code is preserved for anterior and posterior DTCs.
(TIF)

Table S1 Strains used in the analysis.
(DOCX)

Table S2 A/P polarity reversals and D/V migration defects in MIG-14/Wntless, SFRP-1, and/or Netrin signaling component mutants. 1 DTC migration patterns were analyzed by DIC optics in L4 larvae or adults. Numbers represent the percentage of A/P polarity reversals or D/V guidance defects of anterior and posterior DTCs as evidenced by ventralized gonad arms. n = number of gonad arms scored. SE = standard error of the proportion. 2 *mig-14(ga62); unc-5(ev489)* were also analyzed without the *gly-18p::gfp* marker. 3 Most of the progeny of this double mutant strain were inviable (see Results). The escapers were analyzed for DTC migration patterns.
(DOCX)

Table S3 A/P polarity reversals of Wnt receptor mutants, treated or not with *unc-5(RNAi)*. 1 DTC migration patterns were analyzed by DIC optics in L4 larvae or adults. Numbers represent the percentage of anterior and posterior DTC A/P polarity reversals or phase 2 D/V migration failures (i.e., ventralized gonads). n = number of gonad arms scored. SE = standard error of the proportion. 2 D/V guidance defects result from impairing *unc-5* function and reflect the efficacy of the *unc-5(RNAi)* in the population. 3 Analyzed at 25°C, which is a non-permissive temperature for this temperature-sensitive allele.
(DOCX)

Table S4 *unc-5(RNAi)* phenocopies *unc-5* loss-of-function mutations. 1 DTC migration patterns were analyzed by DIC optics in L4 larvae and adults. Numbers represent the percentage of A/P polarity reversals in anterior and posterior. n = number of gonad arms scored. SE = standard error of the proportion.
(DOCX)

Table S5 A/P polarity reversals of Wnt mutants, treated or not with *unc-5(RNAi)*. 1 DTC migration patterns were analyzed by DIC optics in the L4 larvae and adults. Numbers represent the percentage of phase 3 A/P polarity reversals or phase 2 D/V guidance for anterior and posterior DTCs as evidenced by ‘ventralized’ gonad arms. 2 D/V guidance defects result from impairing *unc-5* function and reflect the efficacy of the *unc-5(RNAi)* in the population. 3 *^mom^-2(or85)* is a recessive, non-conditional maternal effect embryonic lethal. Heterozygotes are Unc. Unc hermaphrodites were injected for RNAi, the non-Unc progeny were analyzed. n = number of gonad arms scored. SE = standard error of the proportion.
(DOCX)

Table S6 CAN axon guidance defects in Wnt and/or Netrin signaling component mutants. 1 CAN axon processes were visualized by fluorescence microscopy in L3 larvae to adult stages. All strains contained *gly-18p::gfp* expressed in the CAN neuron. Numbers represent the percentage of CAN axon defects. 2 Incubation temperature. Strains were analyzed at 20°C unless otherwise indicated. n = number of CAN neurons scored. SE = standard error of the proportion.
(DOCX)

Table S7 AVM, PVM or ALM axon guidance defects in mutants of MIG-14/Wntless, EGL-20/Wnt, and Netrin signaling components. 1 mechanosensory neuron axon processes were visualized by fluorescence microscopy in L3 larvae to the adult stage. All strains contained *muIs32[mec-7p::gfp]* or *zdIs5[mec-4p::gfp]*. Numbers represent the percentage of AVM, PVM, or ALM axon guidance defects. 2 Incubation temperature. Strains were analyzed at 20°C unless otherwise indicated. *egl-20(n585)* is reportedly temperature sensitive [33] hence the analysis was carried out at both 20°C and 25°C. 3 *evIs41[mec-7::unc-5; mec-7::lac-z; dpy-20(+)]*. Two independent lines of *unc-5(e53) egl-20(n585); muIs32; evis41* were analyzed. 4 *muIs32* is integrated close to the *mig-14* locus, therefore the analysis of *mig-14/wntless; unc-6/netrin* double mutants and their respective controls was carried out using *zdIs5*. n = number of worms scored. SE = standard error of the proportion.
(DOCX)
