# Supplementary figures and images for: Netrins and Wnts Function Redundantly to Regulate Antero-Posterior and Dorso-Ventral Guidance in C. elegans
Source: PLoS Genet. 2014 Jun 5;10(6):e1004381. doi: 10.1371/journal.pgen.1004381 (PMC4046927; doi:10.1371/journal.pgen.1004381)

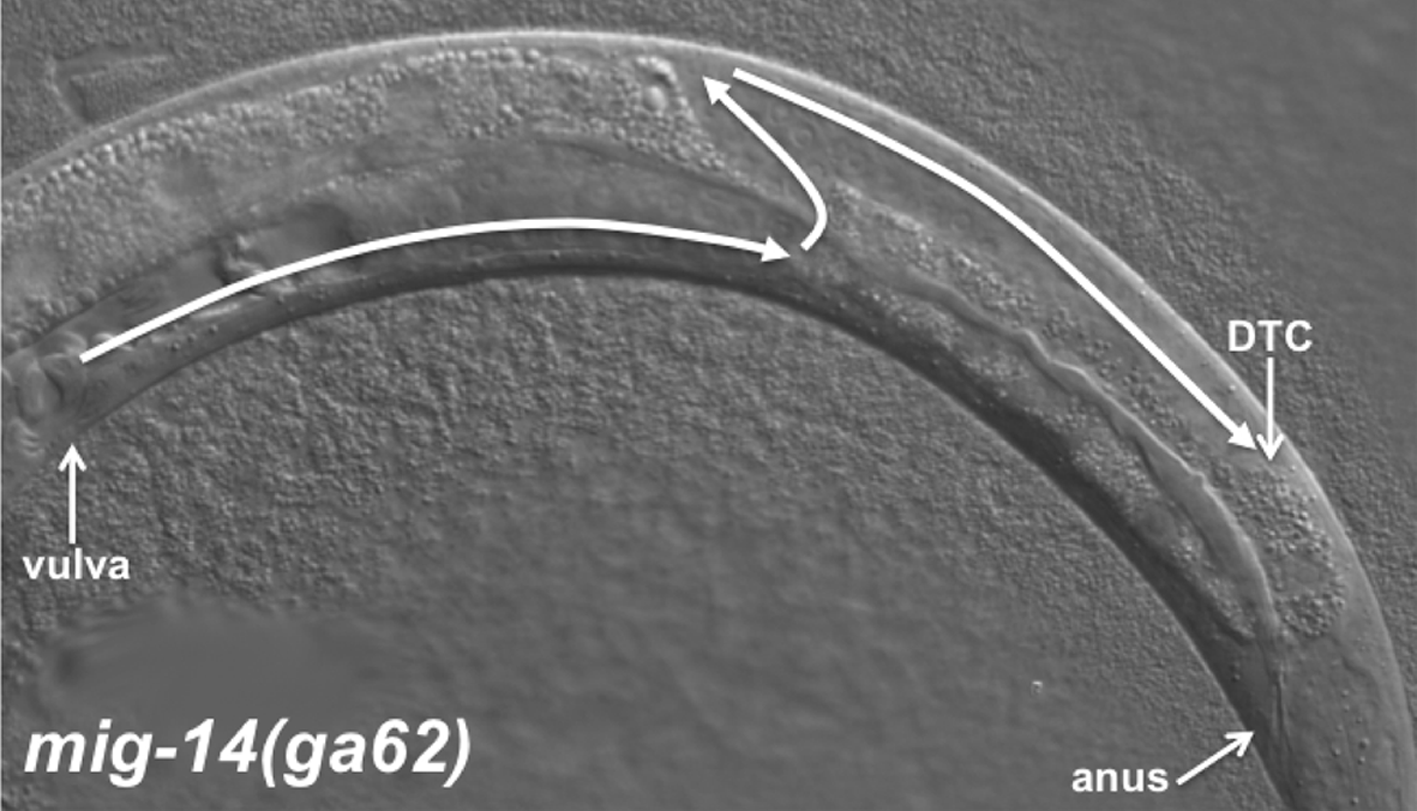

Supplement: Figure S1 — mig-14/wntless displays phase 3 polarity reversals. DIC image shows migration of the posterior DTC. Anterior is left and dorsal is up. L4 stage worm is shown. In some mig-14(ga62) animals the DTC initially reorients back to the mid-body and only subsequently reverses its polarity 180° to migrate away from the mid-body of the animal. (TIF) [file pgen.1004381.s001.tif]

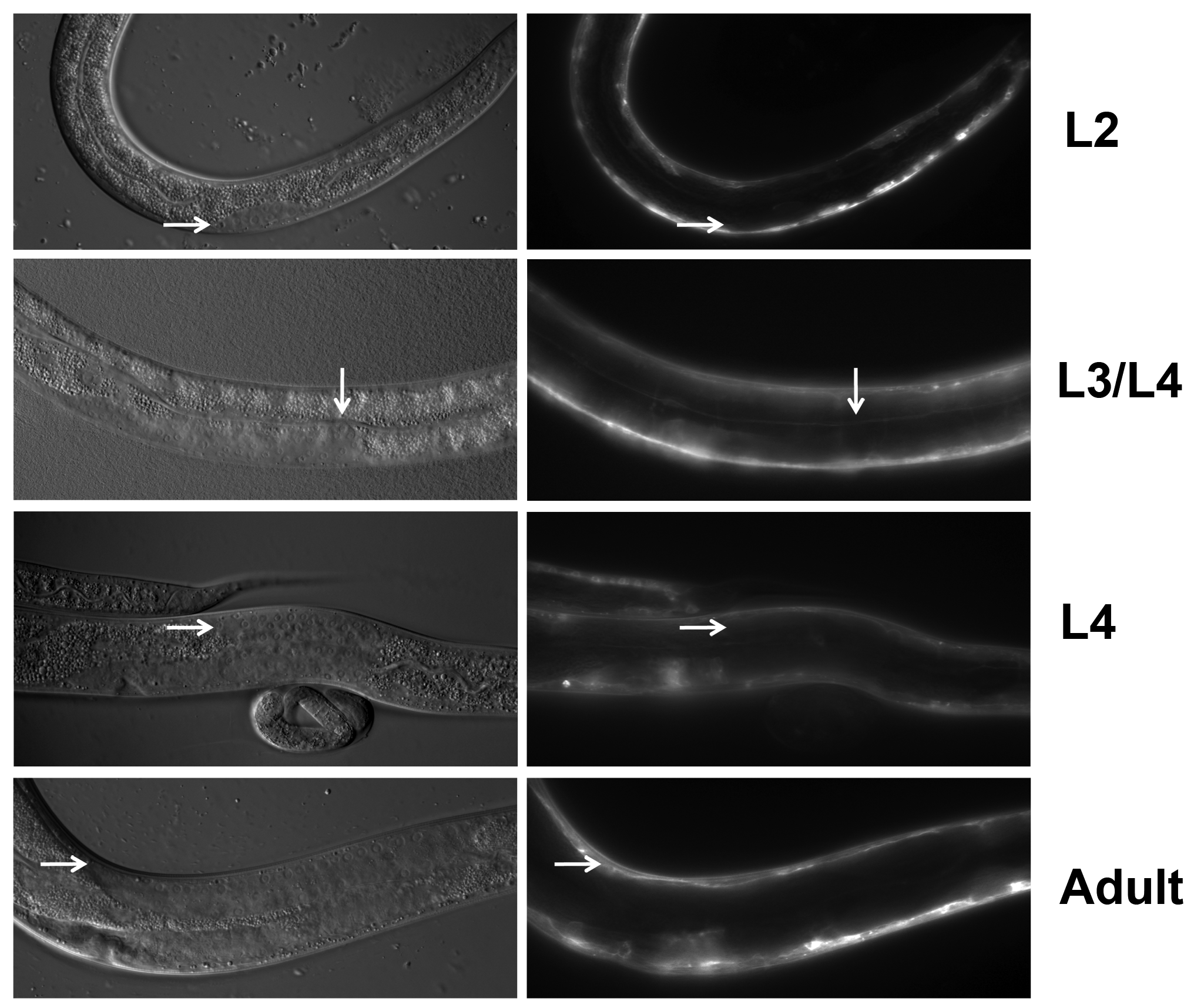

Supplement: Figure S2 — LIN-18 is not detected in the hermaphrodite DTCs throughout development. DIC and fluorescence micrographs of hermaphrodites carrying syIs75, an integrated lin-18::gfp transgene array. Anterior is left and dorsal is up. Arrows mark the DTC. Developmental stage is indicated on the right. L2–L4 represent larval stages preceding the adult stage. (TIF) [file pgen.1004381.s002.tif]

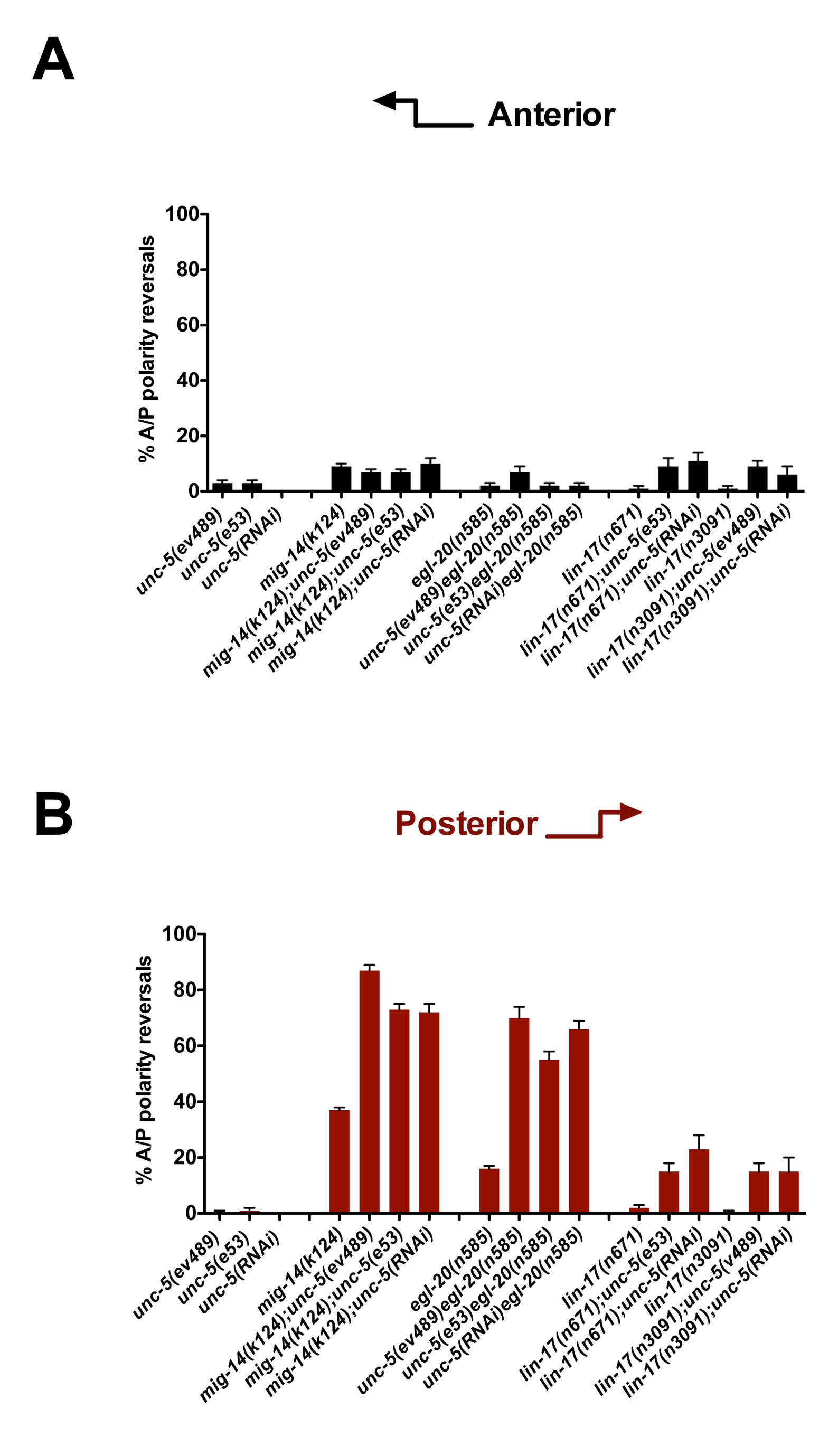

Supplement: Figure S3 — unc-5(RNAi) phenocopies unc-5 loss-of-function mutations. The effects of unc-5(e53) or (ev489) alleles versus unc-5(RNAi) on the frequency of phase 3 A/P polarity reversals in mig-14/wntless, egl-20/wnt or lin-17/frizzled mutants are shown as the percentage of phase 3 A/P polarity reversals for anterior (top panel, black bars) or posterior (bottom panel, red bars) DTCs. The corresponding raw data are presented in Table S4. Error bars indicate the standard error of the sample proportion. (TIF) [file pgen.1004381.s003.tif]

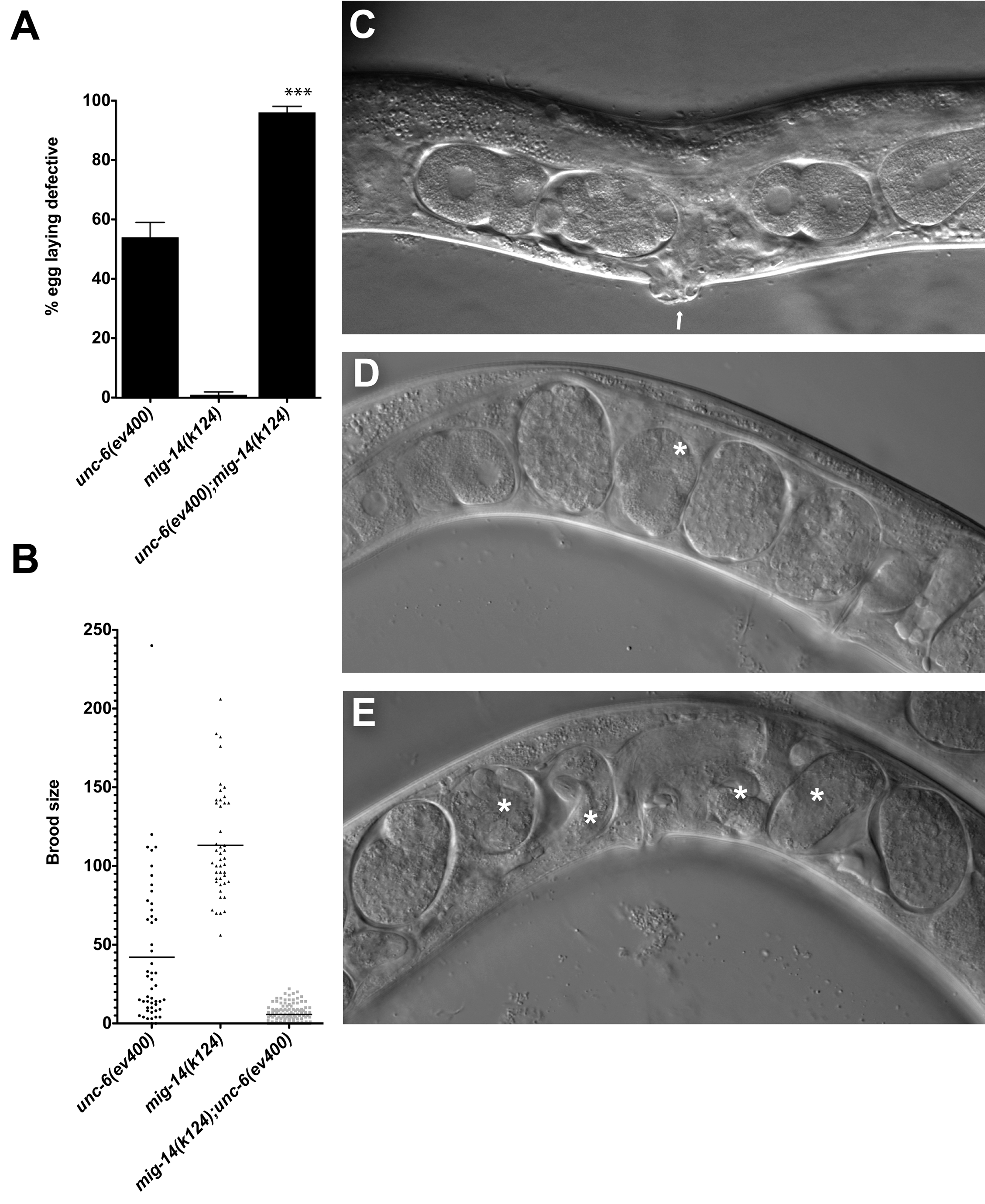

Supplement: Figure S4 — Netrin and Wnt signaling components function redundantly to regulate vulval morphogenesis, vulval function and processes essential for viability. In all panels anterior is left and dorsal is up. (A) Bars represent the percentage of egg laying defects in unc-6(ev400) or mig-14(k124) animals compared to the mig-14(k124); unc-6(ev400) double mutant animals. Error bars indicate standard error of the sample proportion. Comparisons were made to the corresponding single mutant controls. ***P<0.00001. (B) Plot of the individual brood sizes of unc-6(ev400) and mig-14(k124) mutant hermaphrodites compare to mig-14(k124); unc-6(ev400) double mutant hermaphrodites. (C–E) DIC images of mig-14(k124); unc-6(ev400) hermaphrodites. (C) mig-14(k124); unc-6(ev400) double mutants frequently display malformed, protruding vulvae. (D) Arrested, or (E) malformed embryos are frequently observed in the mig-14(k124); unc-6(ev400) hermaphrodite gonads. (TIF) [file pgen.1004381.s004.tif]

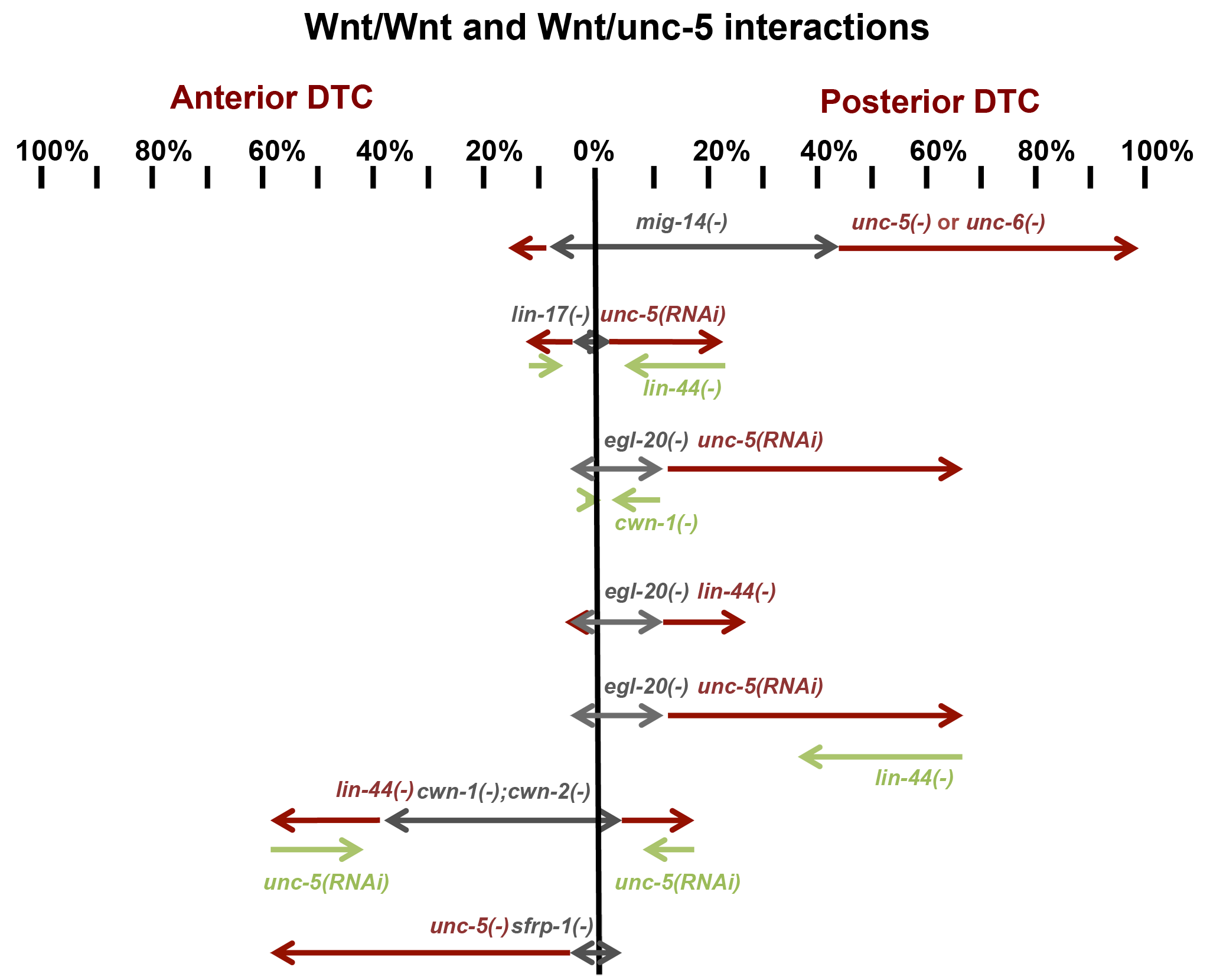

Supplement: Figure S5 — Summary of genetic interactions observed between mutants of different Wnt signaling components or between Wnt components and UNC-5. The extent of each arrow reports the approximate percentage of phase 3 polarity reversals caused by a Wnt signaling component deficit or an unc-5 deficit (color coded). Deficits caused by lof mutations are denoted by (-) and those caused by RNA interference are denoted by (RNAi). The summed extent of the grey and red arrows on the same horizontal line pointing in the same direction represents the enhanced penetrance of the multiple mutant deficits, indicating redundant functions. Green arrows pointing in the reverse direction represent the extent of suppression of the above double mutants by an additional deficit (green), indicating opposing functions. The color code is preserved for anterior and posterior DTCs. (TIF) [file pgen.1004381.s005.tif]
