# Supplementary material for: Netrins and Wnts Function Redundantly to Regulate Antero-Posterior and Dorso-Ventral Guidance in C. elegans
Source: PLoS Genet. 2014 Jun 5;10(6):e1004381. doi: 10.1371/journal.pgen.1004381 (PMC4046927; doi:10.1371/journal.pgen.1004381)
Supplement: Table S1 — Strains used in the analysis. (DOCX) [file pgen.1004381.s006.docx]

| **Strain** | ***C. elegans* gene** | **Encoded product** | **Allelic description** |
| --- | --- | --- | --- |
| **DE60** | unc-119(e2498); dnIs13*[gly-18p::gfp + unc-119(+)]* | GFP reporter for DTCs and CAN | *gly-18p::gfp* is expressed in the DTCs and CAN. Kindly provided by Wendy Johnston and Aldis Krizus |
| **NW2256** | *unc-6(ev400);* dnIs13 | Netrin | Putative null [1] |
| **NW2253** | *unc-40(e1430);* dnIs13 | Netrin receptor (DCC) | Putative null [1] |
| **NW2252** | *unc-5(e53);* dnIs13 | Netrin receptor (UNC-5) | Putative null W283 TAG [2] |
| **NW2254** | *unc-5(ev489);* dnIs13 | Netrin receptor (UNC-5) | Putative null W41 [2] |
| **NW2255** | *unc-5(ev644);* dnIs13 | Netrin receptor (UNC-5) | Hypomorph [3] |
| **NW2244** | *mig-14(ga62);* dnIs13 | Wntless | Strong loss of function. Substitution missense H329N [4] |
| **NW2246** | *mig-14(k124);* dnIs13 | Wntless | Loss of function [5] |
| **RB763** | *cwn-1(ok546)* | Wnt | Putative null. 787 bp deletion deleting exons 2, 3 and majority of 4 [6] |
| **VC636** | *cwn-2(ok895)* | Wnt | Putative null. 906 bp deletion (nucleotide 482-1386) deleting part of exon 2 to 5 [6] |
| **EW72** | *cwn-1(ok546); cwn-2(ok895)* | Wnt; Wnt |  |
| **EW53** | *lin-44(n1792); cwn-1(ok546); cwn-2(ok895)* | Wnt; Wnt; Wnt |  |
| **MT1215** | *egl-20(n585)* | Wnt | Strong loss of function G295A (C99S) [7]. |
| **KN594** | *cwn-1(ok546); egl-20(n585)* | Wnt; Wnt |  |
| **MT5383** | *lin-44(n1792)* | Wnt | Putative null (same over a deficiency). W100 Amber [8]. |
| **KS46** | *lin-44(n1792); egl-20(n585)* | Wnt; Wnt |  |
| **EU365** | *mom-2(or85)V/nT1[unc-?(n754)let-?](IV;V)* | Wnt | Recessive, non-conditional maternal-effect embryonic lethal. Strong allele; missense mutation near N-terminus: L77P. |
| **MT4705** | *lin-17(n671) lin-44(n1792); him-5(e1490)* | Frizzled; Wnt |  |
| **MT1306** | *lin-17(n671)* | Frizzled receptor | Putative null. Ochre (C1345T) [9] |
| **MT8904** | *lin-17(n3091)* | Frizzled receptor | Likely a null [9] |
| **MT2129** | *lin-18(n1051)* | Ryk/Derailed receptor | Putative null. W69 Amber stop [10]. The stop is in the extracellular domain. |
| **CB3303** | *mig-1(e1787)* | Frizzled receptor | Q277ochre stop [11] |
| **MT3148** | *mig-1(n1354)* | Frizzled receptor | Hypomorph |
| **MT3969** | *mig-1(n1652)* | Frizzled receptor | Hypomorph [11] |
| **RB1162** | *cfz-2(ok1201)* | Frizzled receptor | 1175 bp deletion from nt1645 to nt2819. Putative null [6] |
| **NW2245** | *mig-14(ga62); unc-5(ev489);* dnIs13 | Wntless;UNC-5 |  |
| **NW2247** | *mig-14(k124); unc-5(ev489);* dnIs13 | Wntless;UNC-5 |  |
| **NW2248** | *mig-14(k124); unc-5(e53);* dnIs13 | Wntless;UNC-5 |  |
| **NW2250** | *mig-14(k124); unc-5(ev644);* dnIs13 | Wntless;UNC-5 |  |
| **NW2249** | *mig-14(k124); unc-6(ev600);* dnIs13 | Wntless;Netrin | Displayed inviability, could be maintained. The escapers were analyzed. |
| **NW2251** | *unc-40(e1430); mig-14(k124);* dnIs13 | UNC-40/DCC;Wntless |  |
| **PS3720** | *unc-119(ed4); syIs75* | LIN-18::GFP fusion protein | *syIs75[lin-18::gfp + unc-119(+)]* |
| **VC1212** | *sfrp-1(gk554)* | SFRP (Wnt inhibitor) | Putative null, truncates the *sfrp-1* gene upstream of the *CRD and NTR domains*[12] |
| **NW2272** | *egl-20(n585);* dnIs13 | Wnt |  |
| **NW2273** | *unc-5(e53) egl-20(n585);* dnIs13 | UNC-5; Wnt |  |
| **NW2274** | *unc-5(ev489) egl-20(n585);* dnIs13 | UNC-5; Wnt |  |
| **NW2275** | *muIs32; unc-5(e53)* | UNC-5 | *muIs32[mec-7p::gfp + lin-15(+)]. mec-7::gfp* is expressed in touch neurons. |
| **NW2276** | *muIs32; egl-20(n585)* | Wnt |  |
| **NW2277** | *muIs32; unc-5(e53) egl-20(n585)* | UNC-5; Wnt |  |
| **NW2278** | *muIs32; unc-5(ev489) egl-20(n585)* | UNC-5; Wnt |  |
| **NW2292** | *muIs32; unc-5(e53 ) egl-20(n585); evIs41-1* | UNC-5; Wnt | *evIs41[mec-7::unc-5 + mec-7::lacZ + dpy-20(+)]* |
| **NW2293** | *muIs32; unc-5(e53) egl-20(n585); evIs41-2* | UNC-5; Wnt |  |
| **NW2279** | *unc-6(ev400); zdIs5* | Netrin | *zdIs5[mec-4::gfp + lin-15(+) (pSK1)]. mec-4::gfp* is expressed in touch neurons. |
| **NW2280** | *mig-14(k124); zdIs5* | Wntless |  |
| **NW2281** | *mig-14(k124); unc-6(ev400); zdIs5* | Wntless; Netrin |  |
| **NW2288** | *lin-17(n671);* dnIs13 | Frizzled |  |
| **NW2289** | *lin-17(n671);* unc-5(e53); dnIs13 | Frizzled; unc-5 |  |
| **NW2290** | *lin-17(n3091);* dnIs13 | Frizzled |  |
| **NW2291** | *lin-17(n3091);* unc-5(ev489); dnIs13 | Frizzled; unc-5 |  |
| **KS411** | *lin-17(n671); unc-119(e2498); him-5(e1490); mhIs9* | LIN-17::GFP | *mhIs9[lin-17::gfp + unc-119(+)]* [13] |

**Genotyping primers**

| Strain | Forward primer | Reverse primer | Enzymatic digest |
| --- | --- | --- | --- |
| *mig-14(ga62)* | ttgaatggatctccttgacctacc | attgacagcttgccgagtttg | SfaNI |
| *unc-40(e1430)* | ggatagaatcagtgagcagtgccg | gacatgggcttggagcctca | DpnII |
| *egl-20(n585)* | atgcaattcataggtagactgggt | gtcgtcgacttgatggcttg | Bsm-I |
| *evIs41* | aaatgtaaacctgtcatttctgtg | ctcactgtgccggacactcttgtgtc |  |
| *cfz-2(ok1201)* | tcttcggaatggctgcctcggtttggtggg | attccgaaagctcgacaaga |  |

**References**

1. Hedgecock EM, Culotti JG, Hall DH (1990) The unc-5, unc-6, and unc-40 genes guide circumferential migrations of pioneer axons and mesodermal cells on the epidermis in C. elegans. Neuron 4: 61–85.

2. Killeen M, Tong J, Krizus A, Steven R, Scott I, et al. (2002) UNC-5 Function Requires Phosphorylation of Cytoplasmic Tyrosine 482, but Its UNC-40-Independent Functions also Require a Region between the ZU-5 and Death Domains. Dev Biol 251: 348–366. doi:10.1006/dbio.2002.0825.

3. Merz DC, Alves G, Kawano T, Zheng H, Culotti JG (2003) UNC-52/Perlecan affects gonadal leader cell migrations in c. elegans hermaphrodites through alterations in growth factor signaling. Dev Biol 256: 174–187. doi:10.1016/S0012-1606(03)00014-9.

4. Yang P-T, Lorenowicz MJ, Silhankova M, Coudreuse DYM, Betist MC, et al. (2008) Wnt signaling requires retromer-dependent recycling of MIG-14/Wntless in Wnt-producing cells. Dev Cell 14: 140–147. doi:10.1016/j.devcel.2007.12.004.

5. Nishiwaki K (1999) Mutations affecting symmetrical migration of distal tip cells in Caenorhabditis elegans. Genetics 152: 985–997.

6. Zinovyeva AY, Forrester WC (2005) The C. elegans Frizzled CFZ-2 is required for cell migration and interacts with multiple Wnt signaling pathways. Dev Biol 285: 447–461. doi:10.1016/j.ydbio.2005.07.014.

7. Maloof JN, Whangbo J, Harris JM, Jongeward GD, Kenyon C (1999) A Wnt signaling pathway controls hox gene expression and neuroblast migration in C. elegans. Development 126: 37–49.

8. Herman M a, Vassilieva LL, Horvitz HR, Shaw JE, Herman RK (1995) The C. elegans gene lin-44, which controls the polarity of certain asymmetric cell divisions, encodes a Wnt protein and acts cell nonautonomously. Cell 83: 101–110.

9. Sawa H, Lobel L, Horvitz HR (1996) The Caenorhabditis elegans gene lin-17, which is required for certain asymmetric cell divisions, encodes a putative seven-transmembrane protein similar to the Drosophila frizzled protein. Genes Dev 10: 2189–2197.

10. Inoue T, Oz HS, Wiland D, Gharib S, Deshpande R, et al. (2004) C. elegans LIN-18 is a Ryk ortholog and functions in parallel to LIN-17/Frizzled in Wnt signaling. Cell 118: 795–806. doi:10.1016/j.cell.2004.09.001.

11. Pan C-L, Howell JE, Clark SG, Hilliard M, Cordes S, et al. (2006) Multiple Wnts and frizzled receptors regulate anteriorly directed cell and growth cone migrations in Caenorhabditis elegans. Dev Cell 10: 367–377. doi:10.1016/j.devcel.2006.02.010.

12. Harterink M, Kim DH, Middelkoop TC, Doan TD, van Oudenaarden A, et al. (2011) Neuroblast migration along the anteroposterior axis of C. elegans is controlled by opposing gradients of Wnts and a secreted Frizzled-related protein. Development 138: 2915–2924. doi:10.1242/dev.064733.

13. Wu M, Herman MA (2007) Asymmetric localizations of LIN-17/Fz and MIG-5/Dsh are involved in the asymmetric B cell division in C. elegans. Dev Biol 303: 650–662.
