# Supplementary material for: Netrins and Wnts Function Redundantly to Regulate Antero-Posterior and Dorso-Ventral Guidance in C. elegans
Source: PLoS Genet. 2014 Jun 5;10(6):e1004381. doi: 10.1371/journal.pgen.1004381 (PMC4046927; doi:10.1371/journal.pgen.1004381)
Supplement: Table S2 — A/P polarity reversals and D/V migration defects in MIG-14/Wntless, SFRP-1, and/or Netrin signaling component mutants. 1 DTC migration patterns were analyzed by DIC optics in L4 larvae or adults. Numbers represent the percentage of A/P polarity reversals or D/V guidance defects of anterior and posterior DTCs as evidenced by ventralized gonad arms. n = number of gonad arms scored. SE = standard error of the proportion. 2 mig-14(ga62); unc-5(ev489) were also analyzed without the gly-18p::gfp marker. 3 Most of the progeny of this double mutant strain were inviable (see Results). The escapers were analyzed for DTC migration patterns. (DOCX) [file pgen.1004381.s007.docx]

| **Phase 3 polarity reversals:** | **Anterior** | | | | | **Posterior** | | | | |
| --- | --- | --- | --- | --- | --- | --- | --- | --- | --- | --- |
|  | **A/P reversals** | **no turn** | **Total** | **SE** | **n** | **A/P reversals** | **no turn** | **Total** | **SE** | **n** |
| **dnIs13*[gly-18p::gfp]*** | <0.5 | 0 | **0.5** | 0.5 | 223 | 0 | 0 | **0** | 0 | 223 |
| ***mig-14(ga62);* dnIs13** | 6 | 0 | **6** | 2 | 193 | 43 | 1 | **44** | 4 | 193 |
| ***mig-14(ga62); unc-5(ev489);* dnIs13** | 1 | 13 | **14** | 3 | 151 | 3 | 94 | **97** | 1 | 151 |
| ***mig-14(ga62); unc-5(ev489)*^2^** | 0 | 11 | **11** | 3 | 139 | 0 | 96 | **96** | 2 | 139 |
| ***unc-5(ev489);* dnIs13** | 1 | 2 | **3** | 1 | 260 | 0.4 | 0 | **0.4** | 0.4 | 260 |
| ***mig-14(k124);* dnIs13** | 9 | 0.1 | **9** | 1 | 1147 | 37 | 0.2 | **37** | 1 | 1147 |
| ***mig-14(k124); unc-5(ev489);* dnIs13** | 2 | 5 | **7** | 1 | 329 | 1 | 86 | **87** | 2 | 329 |
| ***unc-5(e53);* dnIs13** | 0 | 3 | **3** | 1 | 161 | 1 | 0 | **1** | 1 | 161 |
| ***mig-14(k124); unc-5(e53);* dnIs13** | 2 | 5 | **7** | 1 | 499 | 7 | 66 | **73** | 2 | 499 |
| ***unc-40(e1430);* dnIs13** | 1 | 0 | **1** | 1 | 94 | 2 | 1 | **3** | 2 | 94 |
| ***unc-40(e1430); mig-14(k124*); dnIs13** | 2 | 1 | **3** | 1 | 405 | 14 | 38 | **52** | 3 | 405 |
| ***unc-6(ev400);* dnIs13** | 1 | 2 | **3** | 1 | 151 | 1 | 0 | **1** | 1 | 151 |
| ***mig-14(k124); unc-6(ev400);* dnIs13^3^** | 2 | 11 | **13** | 2 | 233 | 2 | 75 | **77** | 3 | 233 |
| ***unc-5(ev644);* dnIs13** | 2 | 0 | **2** | 1 | 140 | 1 | 0 | **1** | 1 | 140 |
| ***mig-14(k124); unc-5(ev644*)*;* dnIs13**  ***sfrp-1(gk554)***  ***unc-5(RNAi) sfrp-1(gk554)***  ***unc-5(RNAi)*** | 0.5  2  20  0 | 0  0  38  0 | **0.5**  **2**  **58**  **0** | 0.1  1  4  0 | 128  230  189  87 | 71  0.5  1  0 | 12  0  1  0 | **83**  **0.5**  **2**  **0** | 3  0.5  1  0 | 128  230  189  87 |
|  |  |  |  |  |  |  |  |  |  |  |
| **phase 2 D/V defects:** | **Anterior** | | | | | **Posterior** | | | | |
|  | **Ventralized** | **no turn** | **Total** | **SE** | **n** | **Ventralized** | **no turn** | **Total** | **SE** | **n** |
| **dnIs13*[gly-18p::gfp]*** | 0 | 0 | **0** | 0 | 223 | 0 | 0 | **0** | 0 | 223 |
| ***mig-14(ga62*)*;* dnIs13** | 0 | 0 | **0** | 0 | 193 | 0.5 | 1 | **1.5** | 1 | 193 |
| ***mig-14(ga62); unc-5(ev489*)*;* dnIs13** | 48 | 13 | **61** | 4 | 151 | 2 | 94 | **96** | 2 | 151 |
| ***mig-14(ga62); unc-5(ev489)*^2^** | 43 | 11 | **54** | 4 | 139 | 2 | 96 | **98** | 1 | 139 |
| ***unc-5(ev489);* dnIs13** | 27 | 2 | **29** | 3 | 260 | 71 | 0 | **71** | 3 | 260 |
| ***mig-14(k124*)*;* dnIs13** | 0.1 | 0.1 | **0.2** | 0 | 1147 | 0.5 | 0.2 | **1** | 0.5 | 1147 |
| ***mig-14(k124); unc-5(ev489*)*;* dnIs13** | 55 | 5 | **60** | 3 | 329 | 5 | 86 | **91** | 2 | 329 |
| ***unc-5(e53*)*;* dnIs13** | 32 | 3 | **35** | 4 | 161 | 69 | 0 | **69** | 4 | 161 |
| ***mig-14(k124); unc-5(e53*)*;* dnIs13** | 47 | 5 | **52** | 2 | 499 | 10 | 66 | **76** | 2 | 499 |
| ***unc-40(e1430);* dnIs13** | 15 | 0 | **15** | 4 | 94 | 44 | 1 | **45** | 5 | 94 |
| ***unc-40(e1430); mig-14(k124);* dnIs13** | 24 | 1 | **25** | 2 | 405 | 2 | 38 | **40** | 2 | 405 |
| ***unc-6(ev400*)*;* dnIs13** | 42 | 2 | **44** | 4 | 151 | 70 | 0 | **70** | 4 | 151 |
| ***mig-14(k124); unc-6(ev400);* dnIs13^3^** | 54 | 11 | **65** | 3 | 233 | 13 | 75 | **88** | 2 | 233 |
| ***unc-5(ev644);* dnIs13** | 3 | 0 | **3** | 1 | 140 | 11 | 0 | **11** | 3 | 140 |
