# Supplementary material for: Netrins and Wnts Function Redundantly to Regulate Antero-Posterior and Dorso-Ventral Guidance in C. elegans
Source: PLoS Genet. 2014 Jun 5;10(6):e1004381. doi: 10.1371/journal.pgen.1004381 (PMC4046927; doi:10.1371/journal.pgen.1004381)
Supplement: Table S3 — A/P polarity reversals of Wnt receptor mutants, treated or not with unc-5(RNAi). 1 DTC migration patterns were analyzed by DIC optics in L4 larvae or adults. Numbers represent the percentage of anterior and posterior DTC A/P polarity reversals or phase 2 D/V migration failures (i.e., ventralized gonads). n = number of gonad arms scored. SE = standard error of the proportion. 2 D/V guidance defects result from impairing unc-5 function and reflect the efficacy of the unc-5(RNAi) in the population. 3 Analyzed at 25°C, which is a non-permissive temperature for this temperature-sensitive allele. (DOCX) [file pgen.1004381.s008.docx]

| **A/P polarity reversals:** | **Anterior** | | | **Posterior** | | |
| --- | --- | --- | --- | --- | --- | --- |
|  | **A/P reversals** | **SE** | **n** | **A/P reversals** | **SE** | **n** |
| ***wild type*** | 0 | 0 | 100 | 0 | 0 | 100 |
| ***unc-5(RNAi)*** | 0 | 0 | 87 | 0 | 0 | 87 |
| ***lin-17(n3091)*** | 1 | 1 | 107 | 1 | 1 | 107 |
| ***lin-17(n3091); unc-5(RNAi)*** | 6 | 3 | 53 | 15 | 5 | 53 |
| ***lin-17(n671)*** | 2 | 2 | 81 | 2 | 2 | 81 |
| ***lin-17(n671); unc-5(RNAi)*** | 11 | 3 | 86 | 23 | 5 | 86 |
| ***lin-17(n671) lin-44(n1792)*** | 0 | 0 | 139 | 0 | 0 | 139 |
| ***lin-17(n671) lin-44(n1792); unc-5(RNAi)*** | 3 | 2 | 90 | 6 | 3 | 90 |
| ***lin-18(n1051)*** | 0 | 0 | 195 | 0 | 0 | 195 |
| ***unc-5(RNAi); lin-18(n1051)*** | 0 | 0 | 77 | 0 | 0 | 77 |
| ***lin-18(n1051)*^3^** | 1 | 1 | 126 | 1 | 1 | 126 |
| ***unc-5(RNAi); lin-18(n1051)*^3^** | 1 | 1 | 124 | 0 | 0 | 124 |
| ***mig-1(n1652)*** | 0 | 0 | 182 | 0.5 | 0.1 | 182 |
| ***mig-1(n1652); unc-5(RNAi)*** | 0 | 0 | 598 | 0 | 0 | 598 |
| ***mig-1(n1354)*** | 0 | 0 | 148 | 0 | 0 | 148 |
| ***mig-1(n1354); unc-5(RNAi)*** | 0 | 0 | 84 | 0 | 0 | 84 |
| ***mig-1(n1787)*** | 0 | 0 | 175 | 1 | 1 | 175 |
| ***mig-1(n1787); unc-5(RNAi)*** | 1 | 1 | 175 | 0.6 | 0.6 | 175 |
| ***cfz-2(ok1201)*** | 1 | 1 | 122 | 3 | 2 | 122 |
| ***unc-5(RNAi); cfz-2(ok1201)*** | 1 | 1 | 295 | 1 | 1 | 295 |
|  | | | | | | |
| **D/V defects^2^:** | **Anterior** | | | **Posterior** | | |
|  | **Ventralized** | **SE** | **n** | **Ventralized** | **SE** | **n** |
| ***wild type*** | 0 | 0 | 100 | 0 | 0 | 100 |
| ***unc-5(RNAi)*** | 15 | 4 | 87 | 47 | 5 | 87 |
| ***lin-17(n3091)*** | 1 | 1 | 107 | 0 | 0 | 107 |
| ***lin-17(n3091); unc-5(RNAi)*** | 6 | 3 | 53 | 17 | 5 | 53 |
| ***lin-17(n671)*** | 1 | 1 | 81 | 1 | 1 | 81 |
| ***lin-17(n671); unc-5(RNAi)*** | 27 | 5 | 86 | 38 | 5 | 86 |
| ***lin-17(n671) lin-44(n1792)*** | 0.5 | 0.5 | 139 | 0 | 0 | 139 |
| ***lin-17(n671) lin-44(n1792); unc-5(RNAi)*** | 20 | 4 | 90 | 42 | 5 | 90 |
| ***lin-18(n1051)*** | 0 | 0 | 195 | 0 | 0 | 195 |
| ***unc-5(RNAi); lin-18(n1051)*** | 13 | 4 | 77 | 44 | 6 | 77 |
| ***lin-18(n1051)*^3^** | 0 | 0 | 126 | 0 | 0 | 126 |
| ***unc-5(RNAi); lin-18(n1051)*^3^** | 16 | 3 | 124 | 41 | 4 | 124 |
| ***mig-1(n1652)*** | 0 | 0 | 182 | 0.6 | 0.5 | 182 |
| ***mig-1(n1652); unc-5(RNAi)*** | 8 | 1 | 598 | 33 | 2 | 598 |
| ***mig-1(n1354)*** | 0 | 0 | 148 | 0 | 0 | 148 |
| ***mig-1(n1354); unc-5(RNAi)*** | 16 | 4 | 84 | 33 | 5 | 84 |
| ***mig-1(n1787)*** | 0.6 | 0.6 | 175 | 0.6 | 0.6 | 175 |
| ***mig-1(n1787); unc-5(RNAi)*** | 10 | 2 | 175 | 30 | 3 | 175 |
| ***cfz-2(ok1201)*** | 0 | 0 | 122 | 2 | 1 | 122 |
| ***unc-5(RNAi); cfz-2(ok1201)*** | 15 | 2 | 295 | 42 | 3 | 295 |

^\^
