# Supplementary material for: Netrins and Wnts Function Redundantly to Regulate Antero-Posterior and Dorso-Ventral Guidance in C. elegans
Source: PLoS Genet. 2014 Jun 5;10(6):e1004381. doi: 10.1371/journal.pgen.1004381 (PMC4046927; doi:10.1371/journal.pgen.1004381)
Supplement: Table S4 — unc-5(RNAi) phenocopies unc-5 loss-of-function mutations. 1 DTC migration patterns were analyzed by DIC optics in L4 larvae and adults. Numbers represent the percentage of A/P polarity reversals in anterior and posterior. n = number of gonad arms scored. SE = standard error of the proportion. (DOCX) [file pgen.1004381.s009.docx]

| **A/P polarity reversals:** | **Anterior** | | | **Posterior** | | |
| --- | --- | --- | --- | --- | --- | --- |
|  | **A/P reversals** | **SE** | **n** | **A/P reversals** | **SE** | **n** |
| ***unc-5(ev489)*** | 3 | 1 | 260 | 0.5 | 0.5 | 260 |
| ***unc-5(e53)*** | 3 | 1 | 161 | 1 | 1 | 161 |
| ***unc-5(RNAi)*** | 0 | 0 | 87 | 0 | 0 | 87 |
| ***mig-14(k124)*** | 9 | 1 | 1147 | 37 | 1 | 1147 |
| ***mig-14(k124); unc-5(ev489)*** | 7 | 1 | 329 | 87 | 2 | 329 |
| ***mig-14(k124); unc-5(e53)*** | 7 | 1 | 499 | 73 | 2 | 499 |
| ***mig-14(k124); unc-5(RNAi)*** | 10 | 2 | 188 | 72 | 3 | 188 |
| ***egl-20(e585)*** | 2 | 1 | 250 | 16 | 1 | 250 |
| ***unc-5(ev489) egl-20(e585)*** | 7 | 2 | 124 | 70 | 4 | 124 |
| ***unc-5(e53) egl-20(e585)*** | 2 | 1 | 210 | 55 | 3 | 210 |
| ***unc-5(RNAi) egl-20(e585)*** | 2 | 1 | 351 | 66 | 3 | 353 |
| ***lin-17(n671)*** | 1 | 1 | 200 | 2 | 1 | 200 |
| ***lin-17(n671); unc-5(e53)*** | 9 | 3 | 106 | 15 | 3 | 106 |
| ***lin-17(n671); unc-5(RNAi)*** | 11 | 3 | 86 | 23 | 5 | 86 |
| ***lin-17(n3091)*** | 1 | 1 | 229 | 0.5 | 0.5 | 229 |
| ***lin-17(n3091); unc-5(ev489)*** | 9 | 2 | 176 | 15 | 3 | 176 |
| ***lin-17(n3091); unc-5(RNAi)*** | 6 | 3 | 53 | 15 | 5 | 53 |
