# Supplementary material for: Netrins and Wnts Function Redundantly to Regulate Antero-Posterior and Dorso-Ventral Guidance in C. elegans
Source: PLoS Genet. 2014 Jun 5;10(6):e1004381. doi: 10.1371/journal.pgen.1004381 (PMC4046927; doi:10.1371/journal.pgen.1004381)
Supplement: Table S5 — A/P polarity reversals of Wnt mutants, treated or not with unc-5(RNAi). 1 DTC migration patterns were analyzed by DIC optics in the L4 larvae and adults. Numbers represent the percentage of phase 3 A/P polarity reversals or phase 2 D/V guidance for anterior and posterior DTCs as evidenced by ‘ventralized’ gonad arms. 2 D/V guidance defects result from impairing unc-5 function and reflect the efficacy of the unc-5(RNAi) in the population. 3 mom-2(or85) is a recessive, non-conditional maternal effect embryonic lethal. Heterozygotes are Unc. Unc hermaphrodites were injected for RNAi, the non-Unc progeny were analyzed. n = number of gonad arms scored. SE = standard error of the proportion. (DOCX) [file pgen.1004381.s010.docx]

| **A/P polarity reversals:** | **Anterior** | | | **Posterior** | | |
| --- | --- | --- | --- | --- | --- | --- |
|  | **A/P reversals** | **SE** | **n** | **A/P reversals** | **SE** | **n** |
| ***mig-14(k124)*** | **2** | **1** | **147** | **34** | **4** | **147** |
| *mig-14(k124); unc-5(RNAi)* | 10 | 2 | 188 | 72 | 3 | 188 |
| ***egl-20(n585)*** | **2** | **0.5** | **553** | **13** | **1** | **555** |
| *unc-5(RNAi) egl-20(n585)* | 1 | 0.5 | 351 | 66 | 3 | 353 |
| ***cwn-1(ok546); egl-20(n585)*** | **0** | **0** | **190** | **4** | **1** | **190** |
| *cwn-1(ok546); unc-5(RNAi) egl-20(n585)* | 3 | 1 | 245 | 57 | 3 | 244 |
| ***lin-44(n1792)*** | **0.5** | **0.5** | **302** | **0.5** | **0.5** | **302** |
| *lin-44(n1792); unc-5(RNAi)* | 0.5 | 0.5 | 383 | 2 | 1 | 383 |
| ***lin-44(n1792); egl-20(n585)*** | **3** | **1** | **260** | **28** | **3** | **259** |
| *lin-44(n1792); unc-5(RNAi) egl-20(n585)* | 2 | 1 | 241 | 38 | 3 | 242 |
| ***cwn-1(ok546)*** | **1** | **1** | **174** | **0.5** | **0.5** | **174** |
| ***cwn-2(ok895)*** | **2** | **1** | **166** | **0** | **0** | **166** |
| ***cwn-1(ok546); cwn-2(ok895)*** | **38** | **3** | **374** | **3** | **1** | **372** |
| *cwn-1(ok546); cwn-2(ok895) unc-5(RNAi)* | 36 | 3 | 310 | 2 | 1 | 309 |
| ***lin-44(n1792); cwn-1(ok546); cwn-2(ok895)*** | **56** | **2** | **500** | **18** | **2** | **502** |
| *lin-44(n1792); cwn-1(ok546); cwn-2(ok895) unc-5(RNAi)* | 42 | 3 | 289 | 9 | 2 | 293 |
| ***mom-2(or85)*^3^** | **1** | **1** | **175** | **1** | **1** | **175** |
| *unc-5(RNAi); mom-2(or85)* | 1 | 1 | 87 | 0 | 0 | 87 |
|  | | | | | | |
| **D/V defects^2^:** | **Anterior** | | | **Posterior** | | |
|  | **Ventralized** | **SE** | **n** | **Ventralized** | **SE** | **n** |
| ***mig-14(k124)*** | **1** | **1** | **147** | **0** | **0** | **147** |
| *mig-14(k124); unc-5(RNAi)* | 11 | 2 | 188 | 31 | 3 | 188 |
| ***egl-20(n585)*** | **0** | **0** | **553** | **0** | **0** | **555** |
| *egl-20(n585); unc-5(RNAi)* | 7 | 1 | 351 | 49 | 3 | 353 |
| ***cwn-1(ok546); egl-20(n585)*** | **0.5** | **0.5** | **190** | **3** | **1** | **190** |
| *cwn-1(ok546); unc-5(RNAi) egl-20(n585)* | 13 | 2 | 245 | 48 | 3 | 244 |
| ***lin-44(n1792)*** | **1** | **0.5** | **302** | **2** | **1** | **302** |
| *lin-44(n1792); unc-5(RNAi)* | 10 | 2 | 383 | 28 | 2 | 383 |
| ***lin-44(n1792); egl-20(n585)*** | **0** | **0** | **260** | **0.5** | **0.5** | **259** |
| *lin-44(n1792); unc-5(RNAi) egl-20(n585)* | 5 | 1 | 241 | 36 | 3 | 242 |
| ***cwn-1(ok546)*** | **0** | **0** | **174** | **0** | **0** | **174** |
| ***cwn-2(ok895)*** | **0** | **0** | **166** | **0** | **0** | **166** |
| ***cwn-1(ok546); cwn-2(ok895)*** | **0** | **0** | **374** | **0.5** | **0.5** | **372** |
| *cwn-1(ok546); cwn-2(ok895); unc-5(RNAi)* | 20 | 2 | 310 | 27 | 3 | 309 |
| ***lin-44(n1792); cwn-1(ok546); cwn-2(ok895)*** | **0** | **0** | **500** | **1** | **0.5** | **502** |
| *lin-44(n1792); cwn-1(ok546); cwn-2(ok895); unc-5(RNAi)* | 8 | 2 | 289 | 22 | 2 | 293 |
| ***mom-2(or85)*** | **1** | **1** | **175** | **1** | **1** | **175** |
| *unc-5(RNAi); mom-2(or85)* | 18 | 4 | 87 | 52 | 5 | 87 |
