# Supplementary material for: Netrins and Wnts Function Redundantly to Regulate Antero-Posterior and Dorso-Ventral Guidance in C. elegans
Source: PLoS Genet. 2014 Jun 5;10(6):e1004381. doi: 10.1371/journal.pgen.1004381 (PMC4046927; doi:10.1371/journal.pgen.1004381)
Supplement: Table S6 — CAN axon guidance defects in Wnt and/or Netrin signaling component mutants. 1 CAN axon processes were visualized by fluorescence microscopy in L3 larvae to adult stages. All strains contained gly-18p::gfp expressed in the CAN neuron. Numbers represent the percentage of CAN axon defects. 2 Incubation temperature. Strains were analyzed at 20°C unless otherwise indicated. n = number of CAN neurons scored. SE = standard error of the proportion. (DOCX) [file pgen.1004381.s011.docx]

|  | **Temperature**^2^ | **Axon reversal** | **Axon branching** | **Axon stop short** | **Total defects** | **SE** | **n** |
| --- | --- | --- | --- | --- | --- | --- | --- |
| *egl-20(n585)* |  | 0 | 0 | 0 | **0** | 0 | 114 |
| *egl-20(n585)* | *25°C* | 0 | <1 | 0 | **<1** | 0.5 | 188 |
| *unc-5(ev489)* |  | <1 | 1 | <1 | **1** | 1 | 196 |
| *unc-5(ev489)* | *25°C* | <1 | 1 | 0 | **1.5** | 1 | 183 |
| *unc-5(ev489) egl-20(n585)* |  | 5 | 2 | 2 | **9** | 2 | 170 |
| *unc-5(ev489) egl-20(n585)* | *25°C* | 7 | 5 | 1 | **13** | 3 | 100 |
| *unc-5(e53)* |  | 0 | 2 | 0 | **2** | 1 | 173 |
| *unc-5(e53)* | *25°C* | 1 | 4 | 0 | **5** | 2 | 200 |
| *unc-5(e53) egl-20(n585)* |  | 4 | 6 | 7 | **17** | 4 | 111 |
| *unc-5(e53) egl-20(n585)* | *25°C* | 10 | 6 | 1 | **17** | 3 | 211 |
| *unc-6(ev400)* |  | <1 | 3 | 0 | **4** | 1 | 158 |
| *mig-14(k124)* |  | 0 | 0 | 0 | **0** | 0 | 186 |
| *mig-14(k124); unc-6(ev400)* |  | 6 | 11 | 1 | **18** | 4 | 104 |
