# Supplementary material for: Netrins and Wnts Function Redundantly to Regulate Antero-Posterior and Dorso-Ventral Guidance in C. elegans
Source: PLoS Genet. 2014 Jun 5;10(6):e1004381. doi: 10.1371/journal.pgen.1004381 (PMC4046927; doi:10.1371/journal.pgen.1004381)
Supplement: Table S7 — AVM, PVM or ALM axon guidance defects in mutants of MIG-14/Wntless, EGL-20/Wnt, and Netrin signaling components. 1 mechanosensory neuron axon processes were visualized by fluorescence microscopy in L3 larvae to the adult stage. All strains contained muIs32[mec-7p::gfp] or zdIs5[mec-4p::gfp]. Numbers represent the percentage of AVM, PVM, or ALM axon guidance defects. 2 Incubation temperature. Strains were analyzed at 20°C unless otherwise indicated. egl-20(n585) is reportedly temperature sensitive [33] hence the analysis was carried out at both 20°C and 25°C. 3 evIs41[mec-7::unc-5; mec-7::lac-z; dpy-20(+)]. Two independent lines of unc-5(e53) egl-20(n585); muIs32; evis41 were analyzed. 4 muIs32 is integrated close to the mig-14 locus, therefore the analysis of mig-14/wntless; unc-6/netrin double mutants and their respective controls was carried out using zdIs5. n = number of worms scored. SE = standard error of the proportion. (DOCX) [file pgen.1004381.s012.docx]

|  | **Temp**^2^ | **AVM/PVM partially longitudinal** | **AVM/PVM longitudinal** | **AVM/PVM axon polarity reversal** | **AVM/PVM bipolar** | **Total defects** | **SE** | **n** | **ALM bipolar** | **ALM axon polarity reversal** |
| --- | --- | --- | --- | --- | --- | --- | --- | --- | --- | --- |
| *muIs32; egl-20(n585)* |  | 4 | 0 | 1 | 0 | **5** | 2 | 102 | **0** | **0** |
| *muIs32; egl-20(n585)* | 25°C | 4 | 3 | 0 | 0 | **7** | 2 | 116 | **1** | **0** |
| *muIs32; unc-5(ev489) egl-20(n585)* |  | 3 | 4 | 3 | 2 | **12** | 3 | 120 | **2** | **3** |
| *muIs32; unc-5(ev489) egl-20(n585)* | 25°C | 1 | 9 | 4 | 5 | **19** | 4 | 75 | **0** | **0** |
| *muIs32; unc-5(e53)* |  | 3 | 0 | 1 | 0 | **4** | 2 | 132 | **0** | **0** |
| *muIs32; unc-5(e53)* | 25°C | 1 | 3 | 1 | 0 | **5** | 2 | 116 | **0** | **0** |
| *muIs32; unc-5(e53) egl-20(n585)* |  | 4 | 15 | 4 | 4 | **27** | 4 | 135 | **1** | **1** |
| *muIs32; unc-5(e53) egl-20(n585)* | 25°C | 6 | 19 | 3 | 9 | **37** | 4 | 125 | **3** | **0** |
| *muIs32; unc-5(e53) egl-20(n585)* | 25°C | 6 | 25 | 2 | 8 | **41** | 4 | 177 | **1** | **0** |
| *muIs32; unc-5(e53) egl-20(n585); evIs41-1*^3^ | 25°C | 8 | 7 | 1 | 4 | **20** | 4 | 106 | **0** | **0** |
| *muIs32; unc-5(e53) egl-20(n585); evIs41-2*^3^ | 25°C | 4 | 7 | 0 | 9 | **20** | 4 | 116 | **0** | **0** |
| *unc-6(ev400); zdIs5*^4^ |  | 4/1 | 24/17 | 2/1 | 1/4 | **31/23** | 4/4 | 128 | **0** | **0** |
| *mig-14(k124); zdIs5* |  | 1/1 | 0 | 0 | 0 | **1/1** | 1/1 | 112 | **6** | **0** |
| *mig-14(k124); unc-6(ev400); zdIs5* |  | 10/6 | 42/48 | 6/15 | **2/0** | **60/69** | 7/6 | 52 | **0** | **4** |
